# Avian Migration-Mediated Transmission and Recombination Driving the Diversity of Gammacoronaviruses and Deltacoronaviruses

**DOI:** 10.1093/molbev/msaf045

**Published:** 2025-02-18

**Authors:** Yuting Xu, Yelin Han, Panpan Xu, Siyu Zhou, Peng Zhao, Yuyang Wang, Jie Hu, Min Ma, Zirong Li, Shunqi Bo, Chenyao Zhao, Lei Ji, Yue Yuan, Wenliang Zhao, Jianwei Wang, Qi Jin, Guimei He, Zhiqiang Wu

**Affiliations:** School of Life Sciences, East China Normal University, Shanghai, PR China; NHC Key Laboratory of Systems Biology of Pathogens, State Key Laboratory of Respiratory Health and Multimorbidity, National Institute of Pathogen Biology, Chinese Academy of Medical Sciences & Peking Union Medical College, Beijing, PR China; NHC Key Laboratory of Systems Biology of Pathogens, State Key Laboratory of Respiratory Health and Multimorbidity, National Institute of Pathogen Biology, Chinese Academy of Medical Sciences & Peking Union Medical College, Beijing, PR China; NHC Key Laboratory of Systems Biology of Pathogens, State Key Laboratory of Respiratory Health and Multimorbidity, National Institute of Pathogen Biology, Chinese Academy of Medical Sciences & Peking Union Medical College, Beijing, PR China; NHC Key Laboratory of Systems Biology of Pathogens, State Key Laboratory of Respiratory Health and Multimorbidity, National Institute of Pathogen Biology, Chinese Academy of Medical Sciences & Peking Union Medical College, Beijing, PR China; NHC Key Laboratory of Systems Biology of Pathogens, State Key Laboratory of Respiratory Health and Multimorbidity, National Institute of Pathogen Biology, Chinese Academy of Medical Sciences & Peking Union Medical College, Beijing, PR China; School of Life Sciences, East China Normal University, Shanghai, PR China; School of Life Sciences, East China Normal University, Shanghai, PR China; Shanghai Forestry Station, Shanghai, PR China; Shanghai Landscaping & City Appearance Administrative Bureau, Shanghai, PR China; Shanghai Forestry Station, Shanghai, PR China; Shanghai Forestry Station, Shanghai, PR China; Shanghai Chongming Dongtan Nature Reserve Administration Center, Shanghai, PR China; NHC Key Laboratory of Systems Biology of Pathogens, State Key Laboratory of Respiratory Health and Multimorbidity, National Institute of Pathogen Biology, Chinese Academy of Medical Sciences & Peking Union Medical College, Beijing, PR China; NHC Key Laboratory of Systems Biology of Pathogens, State Key Laboratory of Respiratory Health and Multimorbidity, National Institute of Pathogen Biology, Chinese Academy of Medical Sciences & Peking Union Medical College, Beijing, PR China; NHC Key Laboratory of Systems Biology of Pathogens, State Key Laboratory of Respiratory Health and Multimorbidity, National Institute of Pathogen Biology, Chinese Academy of Medical Sciences & Peking Union Medical College, Beijing, PR China; School of Life Sciences, East China Normal University, Shanghai, PR China; Institute of Eco-Chongming (IEC), East China Normal University, Shanghai, PR China; Shanghai Institute of Wildlife Epidemics, East China Normal University, Shanghai, PR China; NHC Key Laboratory of Systems Biology of Pathogens, State Key Laboratory of Respiratory Health and Multimorbidity, National Institute of Pathogen Biology, Chinese Academy of Medical Sciences & Peking Union Medical College, Beijing, PR China; Key Laboratory of Pathogen Infection Prevention and Control (Ministry of Education), National Institute of Pathogen Biology, Chinese Academy of Medical Sciences & Peking Union Medical College, Beijing, PR China; School of Population Medicine and Public Health, Chinese Academy of Medical Sciences & Peking Union Medical College, Beijing, PR China

## Abstract

In the wake of pandemics like COVID-19, which have zoonotic origins, the role of wildlife as reservoirs for emerging infectious diseases has garnered heightened attention. Migratory birds, traversing continents, represent a potent but under-researched vector for the spread of infectious diseases, including novel coronaviruses. This study delves into the genetic diversity and transmission dynamics of coronaviruses in migratory birds, presenting pivotal findings. From April 2019 to April 2023, we screened 5,263 migratory bird samples collected from Shanghai, China, identifying 372 coronavirus-positive samples belonging to five avian-related coronavirus subgenera and subsequently obtaining 120 complete genome sequences. To facilitate further research with a global perspective, the study curated all available 19,000 avian-associated coronaviruses and expanded the original 12 species to 16, including three novel coronavirus species identified in our study and one re-classified species from the public domain. The study illuminates the intricate genetic evolution and transmission dynamics of birds-related coronaviruses on a global scale. A notable aspect of our research is the identification of complex recombination patterns within the spike protein across different virus species and subgenera, highlighting migratory birds as a reservoir of coronavirus. Notably, the coronaviruses found in migratory birds, predominantly from the orders Anseriformes, Charadriiformes, and Pelecaniformes, with domestic ducks from Anseriformes playing a key role in bridging the transmission of coronaviruses between migratory and non-migratory birds. These findings reveal the genetic and recombination characteristics of coronaviruses in migratory birds, emphasizing the critical role of ecologically pivotal bird species in coronavirus transmission and genetic diversity shaping.

## Introduction

Since the beginning of the 21st century, the world has witnessed multiple outbreaks of infectious diseases caused by coronaviruses (CoVs), including Severe Acute Respiratory Syndrome Coronavirus (SARS-CoV), Middle East Respiratory Syndrome Coronavirus (MERS-CoV), and SARS-CoV-2 (Ksiazek et al. 2003; Zaki et al. 2012; Zhou et al. 2018; Zhou et al. 2020). These events have resulted in numerous human fatalities and are gradually becoming a One-Health concern. In addition to impacting human health, CoVs also had devastating consequences on livestock and poultry farming, causing gastrointestinal and respiratory illnesses such as porcine deltacoronavirus (PDCoV) in pigs and infectious bronchitis virus (IBV) in chickens, leading to significant economic losses.

Commonly referred to CoVs belong to the subfamily *Orthocoronavirinae*, which is divided into four genera, *Alphacoronavirus* (AlphaCoV), *Betacoronavirus* (BetaCoV), *Gammacoronavirus* (GammaCoV), and *Deltacoronavirus* (DeltaCoV) (https://ictv.global.). AlphaCoV and BetaCov are most commonly infecting bats, which is thought to be the ancestral host of these two genera, while DeltaCoV and GammaCov mostly infect birds(Cui, Li and Shi 2019; Latinne et al. 2020; Wille and Holmes 2020). Although current research on CoVs focuses on bats, the potential harm of genetically diverse CoVs carried by birds should not be overlooked. Some subgenera within GammaCoV and DeltaCoV have gradually obtained the ability to infect mammals. The subgenus *Cegacovirus* of GammaCoVs can infect marine mammals, such as whales and dolphins. PDCoV as an emerging member of DeltaCoVs, originally circulating among migratory birds such as sparrows and quails, has successfully crossed species barriers to infect domestic pigs and spread globally (Woo et al. 2012). Remarkably, PDCoV also infected three Haitian children. The symptoms were an acute undifferentiated febrile illness. This emphasizes the potential risk of transmission of CoVs to mammals, including humans (Lednicky et al. 2021). This phenomenon reveals the potential risk of transmission of CoVs to mammals, emphasizing the imperative for further research into the diversity and dynamics of CoVs carried by birds. Furthermore, spillover of wild bird CoVs to poultry can sometimes lead to severe mortality and subsequently cause important economic losses (Suryaman et al. 2019).

Migratory birds, as significant natural reservoirs for CoVs, provide a unique pathway for the transmission of these viruses through their annual cross-border and transcontinental migrations (Viana, Santamaria and Figuerola 2016). The migratory routes of these birds span the globe(Olson et al. 2023), connecting different ecosystems, thereby offering viruses a wide geographical distribution and a diverse host population. The global spread of highly pathogenic avian influenza provides ample evidence of its potential for interspecies transmission, including cows, cats, dogs, foxes, seals, and even humans. This cross-geographical and virus transmission patterns, coupled with the high genetic variability of CoVs, make their evolutionary dynamics, host adaptability, and potential public health impacts warrant considerable attention. Furthermore, climate change might unpredictably change the migration patterns, and habitat reductions due to human activities exacerbate the risk of interactions between sympatric wild birds and poultry. All those human made changes may further exacerbate the threat posed by CoVs carried by migratory birds to human societies and ecosystems (Mora et al. 2022). In response to these challenges, actively conducting research on migratory birds can enhance our knowledge of the genetic diversity and transmission dynamics of CoVs that could be aid in risk estimation and strategic design for control and prevention of future zoonotic-related infectious diseases.

This study conducted sampling over four consecutive years at Shanghai, China one of the most significant stopover points for migratory birds (Zhou et al. 2016). Combined with metagenomic sequencing technology, the identification and whole-genome acquisition of CoVs in migratory birds were accomplished. To conduct further research on the diverse niches of CoVs carried by migratory birds, we further collected and curated all publicly available viruses within GammaCoVs and DeltaCoVs to build a sequence database of avian-associated CoVs. The research, integrated self-identified CoVs with public datasets on a global scale, collectively unveiled the genetic evolution, host range, transmission patterns.

## Materials and Methods

### Ethics Approval and Biosafety

Wild birds were captured using mist nets or clap traps and sampled with the permission and approval of the Shanghai Wild Life Conservation and Management Office (2019–2023 [32], [54], [64], [144]). After sampling, all birds were released. All experiments were conducted in a biosafety level 2 (BSL-2) laboratory at East China Normal University.

### Sample Collection and Processing

From April 2019 to April 2023, a total of 5,263 samples from 95 species of migratory birds, spanning 14 orders and 28 families and 48 genera, were collected in Shanghai, including Chongming Dongtan wetland (31°25′ −31°38′N, 121°53′ −122°04′ E), Jiuduansha wetland (31°06′ to 31°14′ N, 121°46′ to 122°15′ E) and the Nanhui Dongtan wetland (30°51′ to 31°06′ N, 121°50′ to 121°51′ E). These samples included 3,305 swab samples and 1,803 fecal samples from live birds, in addition to 155 tissue samples obtained from deceased birds encountered during rescue patrols in the field. Upon the capture of each bird, it was immediately assigned a number, and key information such as species, sex (if identifiable), capture location, and time was meticulously recorded. Swab samples were mainly collected from the cloacal region of the birds. All samples were transported to the laboratory at 4 °C immediately after collection and then stored at −80 °C in an ultra-low temperature freezer to ensure their biological integrity and the reliability of subsequent experiments. Bird species identification was conducted by experienced ornithologists based on morphological characteristics, calls, and ecological behaviors. Furthermore, every collected sample was rigorously documented with the sampling time and species information, ensuring the accuracy and validity of data analysis and subsequent research.

### Distribution Maps Drawn From Avian Observation Data

This study compiled a comprehensive catalog of migratory birds carrying CoVs by sourcing a list of migratory birds from Birdlife (https://www.birdlife.org/), combined with a catalog of migratory birds known to carry CoVs from public databases, and a catalog of birds identified for the first time in this study as carriers of the virus. Observational geographical locations (latitude and longitude data) of these birds carrying CoVs were downloaded through GBIF (https://www.gbif.org/occurrence/search). Using the migratory bird list from BirdLife, we downloaded corresponding observation points for these species from GBIF. Data without precise species identification or latitude/longitude coordinates were excluded. The remaining data were organized into a table with three columns: species, longitude, and latitude, for further processing in ArcGIS software. Using ArcGIS software, the observation data of migratory birds carrying CoVs was projected onto a world map and converted from point data into raster data. The raster distribution data for each species was then combined using the mosaic tool to generate a map of richness. This study ultimately produced a map showing the richness of all migratory birds carrying CoVs and the distribution richness of the sampled migratory birds carrying CoVs.

### Rarefaction Analysis and PCA

This study performed a rarefaction analysis, finely processing the annually and monthly collected sample data with the vegan package in R version 4.2.3. By incrementally increasing the sample size, this method simulated the dynamic changes in the number of species types as the sample volume increased, thereby constructing rare curves for each year. This analysis took into account the differences in annual and monthly sampling intensities, allowing for precise observation of the relationship between sample volume and species discovery rate, especially the change in the growth trend of species types after the sample volume increased to a certain stage, thereby effectively revealing the impact of sampling strategies on the depth of species diversity research. Considering the seasonality of bird migration, where birds typically migrate south in autumn and winter and start to return north in spring, we counted the number of samples and corresponding species from October to February and from March to September each year, constructing dilution curves for presentation respectively.

To explore the seasonal variations and interannual differences in species sampling numbers, this study conducted PCA using R version 4.2.3, analyzing the species sampling data from October to February and March to September across different years. Notably, this research incorporated a data transformation method based on Hellinger distance, which involves square root transformation of count data, effectively reducing data skewness and improving the comparability of diversity indices, thereby enhancing the adaptability and accuracy of PCA analysis in handling ecological count data. In essence, this transformation method improves the stability and reliability of the analysis by altering the scale of the data. Furthermore, this study applied a circle clustering method with a 95% confidence interval to identify significant clustering patterns within the data. The clustering analysis not only took into account the grouping of data but also aimed to present information from multiple perspectives, providing a multidimensional method to analyze the seasonal distribution of species. For ungrouped data, independent clustering was utilized, offering a unique perspective on the sampling patterns for each seasonal segment of the year. To comprehensively explore the seasonal patterns of species diversity and their interannual changes, the data were grouped by season, with the data from October to February and March to September being treated as separate groups. These were marked with different colors, visually demonstrating the dynamic changes in seasonal patterns across years.

### Nucleic Acid Extraction From Samples

Initially, the samples were subjected to a preprocessing step. In this study, samples were centrifuged at 12,000 × *g* for 10 min to remove larger impurities, followed by filtration of the supernatant through a 0.45 μm polyvinylidene difluoride filter (Millipore, Germany). The filtrate, equilibrated with Hank's balanced salt solution, was then centrifuged using an ultracentrifuge at 4 °C, 100,000 × *g* for 4 h. After centrifugation, the supernatant was discarded, and the enriched sample at the bottom of the ultracentrifuge tube was resuspended in 106 μl of Hank's solution. The suspension was thoroughly vortexed and transferred to a new centrifuge tube for further use. Subsequently, the 106 μl resuspended enriched sample was digested with Turbo DNase, benzonase, RNase One, and 10*DNase buffer, with the digestion reaction incubated at 37 °C for 2 h. Finally, total viral RNA was extracted from the sample supernatant using the QIAamp Viral RNA Mini Kit. Single-stranded cDNA was synthesized using the primer K-8N and a Superscript IV system (Invitrogen, USA), which was used for subsequent screening and identification of CoVs. Detailed methodologies can be referenced in previous articles (Wu et al. 2012).

### Screening and Identification of CoVs and High-Throughput Sequencing

In this study, two sets of screening primers specifically designed for CoVs were employed to conduct a comprehensive survey of 5,263 migratory bird samples, resulting in the identification of 372 coronavirus-positive specimens. The first set of primers targeted the four genera of CoVs (OutF_5′-C CAARTTYTAYGGHGGITGG-3′; InF_5′- GGTTGGGAYTAYCCHAARTGTGA-3′; R_5′- TGTTGIGARCARAAYTCATGIGG-3′) (Xiu et al. 2020), while the second primer set, developed in-house, aimed at the universal detection of CoVs specifically within the DeltaCoV and GammaCoV genera (OutF_5′-GGBTGGGAYTAYCCBAA RTG-3′; InF_5′- GGTTGGGAYTAYCCYAARTGTGA-3′; OutR_5′- TGYTGTSWR CARAAYTCRTG-3′; InR_5′- ACACCRTCRTCAGAYARDATCAT-3′). Through this process, we compiled statistics on the number of coronavirus-positive samples detected in each species. To visually present these data, we utilized the pheatmap (version 1.0.12), vegan (version 2.6.4) packages in R version 4.2.3 to generate corresponding graphs. This approach allowed us to illustrate the pattern of coronavirus-positive sample distribution across different bird species, enabling us to clearly observe the infection status of CoVs in the migratory bird population and their potential transmission trends.

For the cDNA of samples that tested positive for coronavirus in the screening, ds DNA was obtained using Klenow fragment (NEB, USA), and the PCR products were randomly amplified using primer K. Magnetic beads (Beckman Coulter, USA) were used for the purification of PCR products, followed by the construction of nucleic acid libraries with magnetic beads (Beckman Coulter, USA) for metagenomic sequencing.

### High-Throughput Sequencing Data Processing

Sequencing reads, after quality control, were analyzed by alignment with NCBI's non-redundant nucleotide database (NT) and the non-redundant protein database (NR) using BLASTx. Subsequently, the MEGAN6 (Huson et al. 2016) software utilized the best BLAST scores (E score <10^−5^) to parse and extract taxonomic information of the matched reads. For the assembly of CoVs, we chose the megahit v1.2.9 (Li et al. 2015) software with default settings, using the extracted and assembled contigs as a reference to guide PCR screening verification and subsequent sequencing efforts. We designed nested specific sequence primers to amplify parts of the genome and used the ABI3500 DNA analyzer (Applied Biosystems, USA) for Sanger sequencing of PCR products. The sequences were organized using Geneious Prime software (version 2022.2.2), ultimately obtaining 120 complete full genome sequences.

### Genome Structure Annotation and Classification

For the obtained viral genome sequences, we employed the “Annotate & Predict” function in Geneious Prime software (version 2022.2.2), based on our own rigorously curated and standardized database, to annotate the genome sequences through the “Annotate from Database” feature. Manual corrections were made post-annotation to ensure accuracy. Based on the annotated sequences, we extracted five conserved regions of CoVs, namely 3CLpro, NiRAN, RdRp, ZBD, and HEL1. Using these five conserved regions, we constructed a taxonomic phylogenetic tree according to the ICTV standards and aligned the concatenated sequences of the five conserved regions with the refseqs of each subgenus using MegAlign (DNA Star package Lasergene v.7.0.1). According to ICTV standards, sequences with less than 90% similarity in the conserved regions and reference genomes were considered as potentially new virus species, named DuCoV_NL1, SandCoV_NL2, DuCoV_NL3 and GaCoV_NL4 in this study.

Furthermore, for different virus species within *Deltacoronavirus* and *Gammacoronavirus*, we selected reference sequences to represent the genome structure of these virus species. The GenBank files of reference genomes were imported into SnapGene 5.3.1 software (http://www.snapgene.com), and through manual adjustments, we achieved the visualization of the genome structure.

### Recombination Analysis

For investigating recombination events, we employed the RDP5 software (Martin and Rybicki 2000) to analyze recombination in CoVs belonging to the *Gammacoronavirus* and *Deltacoronavirus* genera. During this process, we used seven different methods to detect potential recombination events, specifically RDP (Martin and Rybicki 2000), GENECONV (Padidam, Sawyer and Fauquet 1999), BOOTSCAN (Martin et al. 2005), MAXCHI (Smith 1992), CHIMAERA (Posada and Crandall 2001), SISCAN (Gibbs, Armstrong and Gibbs 2000), and 3SEQ (Boni, Posada and Feldman 2007). An event was considered a true recombination only if all of these methods identified it. Additionally, we cross-verified the recombination events by examining phylogenetic trees and the statistical outputs from RDP5, ensuring the final confirmation of valid recombination events. All analyses were performed using default settings, except for setting the sequences as linear. To ensure the accuracy of the inferred recombination events, we manually examined the approximate recombination sequences and breakpoints inferred from the detected events, based on the phylogenetic trees and recombination signal analysis features in RDP5, making necessary adjustments. We further analyzed breakpoints distribution to detect potential recombination hot- or cold-spots. For this, we used the Breakpoint Distribution Plot, where the black line represents the number of breakpoints within 200 nucleotides across the genome (x-axis). The light and dark gray shaded regions represent the 95% and 99% confidence intervals for breakpoint clustering under random recombination.

To visually display the clustering trends of coronavirus samples in different genomic regions, In this study, sequences from our database were initially aligned utilizing MAFFT v7.47525 (Katoh and Standley 2013) software. Following this, the optimal model for constructing phylogenetic trees was identified using IQ-Tree (Minh et al. 2020). Key feature data, including clustering information and genetic distances, were then extracted. These data were subsequently used as input for dimensionality reduction and visualization using the t-SNE technique(Van der Maaten and Hinton 2008). By implementing t-SNE dimensionality reduction with the Rtsne package in R version 4.2.3 and adjusting algorithm parameters such as perplexity and learning rate, we optimized the presentation of data in two-dimensional space. This approach clearly revealed the clustering relationships between samples in low-dimensional space, effectively unveiling the genetic relationships among coronavirus samples across different gene regions and achieving an intuitive display of genetic similarities between samples. Additionally, to more comprehensively reflect the recombination scenarios between different genomic segments in CoVs of the *Gammacoronavirus* and *Deltacoronavirus* genera, we selected key virus strains for recombination analysis using SimPlot software (Samson, Lord and Makarenkov 2022). Through SimPlot, we input specific virus strain sequence data and set appropriate sliding window sizes and steps to obtain a graphical display of sequence similarity.

### Sequence Data Collection

To comprehensively retrieve known sequences from public databases, we conducted an initial search in GenBank using the keyword combination (((Orthocoronavirinae) NOT ((Alphacoronavirus) OR Betacoronavirus))) AND ((Deltacoronavirus) OR Gammacoronavirus). This search yielded 16,491 GenBank records as of May 24, 2024, which were subsequently stored on our local system. Comprehensively summarizing these known viral sequences maximizes our understanding of the diversity and ecological characteristics of avian-associated CoVs and facilitates effective surveillance to monitor potential transmission for future emerging zoonotic diseases control and prevention. However, similar to issues identified in our previous study ZOVER, we noted a substantial number of related GenBank records with inaccuracies or incomplete metadata and genome annotations, significantly hindering further data reuse and mining. To address this issue, we meticulously performed a systematic review of published records and literature to construct an online data resource on the genetic diversity, spatiotemporal distribution, and host specificity of DeltaCoVs and GammaCoVs. The database currently contains CoVs from various animal species globally. Furthermore, building upon our previously released platform ZOVER, a set of visualization utilities, tailored exclusively for avian-associated CoVs investigation, was designed to deliver easy browsing and analytical support, including an automatic pipeline for sequence annotation.

### Phylogenetic Evolutionary Analysis and Sequence Consistency Comparison

Initially, sequence alignment was performed using MAFFT v7.475 software, followed by both the determination of the optimal model and the construction of the phylogenetic tree using IQ-Tree 2 software (Letunic and Bork 2021). Then we elaborately annotated via the Interactive Tree Of Life (itol) software by adding detailed information such as labels, subgenera, and species. Additionally, consistency among the sequences was calculated using Geneious Prime software for subsequent analysis.

### Ancestral Host Reconstruction

For the ancestral host reconstruction, we utilized complete S protein sequences from both our study and GenBank, excluding any sequences lacking complete sampling time or host information to ensure the analysis's accuracy. Additionally, we excluded S protein sequences involved in recombination through recombination detection. Sequence alignment was accomplished through MAFFT v7.475, while the construction of the phylogenetic tree was based on the optimal model using IQtree2 software. We found temporal signals detected by TempEst (Rambaut et al. 2016). In subsequent Bayesian analyses, the best-fit model GTR + F + I + G4 was chosen based on the Bayesian Information Criterion (BIC), with a strict clock model and a constant size model as a coalescent tree prior in BEAST v1.10.4 (Drummond and Rambaut 2007). The number of iterations for BEAST runs was the square of the number of sequences multiplied by 3,000. The convergence of the chains was assessed using Tracer v1.7.1 (Rambaut et al. 2018), with our data's Effective Sample Size (ESS) values exceeding 200. The tree files generated by BEAST were annotated using TreeAnnotator v1.10.4 (Helfrich et al. 2018), setting a 10% burn-in. The resulting maximum clade credibility (MCC) tree was further analyzed and visualized using FigTree software (Rambaut 2018). During the annotation process, FigTree was used to apply informative labels, colors, and other visual cues to the tree's nodes and branches, aiming to highlight significant phylogenies, branches, and other key features within the tree structure.

### Haplotype Network Construction

Based on our database, each sequence was annotated with its corresponding host genus and aligned using MAFFT software, saved in NEX format. The haplotype file was generated using DnaSP software (Rozas et al. 2017). The haplotype network was constructed using the TCS method under the network function in PopART software (Leigh, 2015), with some major hosts at the order level marked and silhouettes labeled nearby.

## Results

### Sampling of Migratory Birds in Shanghai

**Fig. 1.**
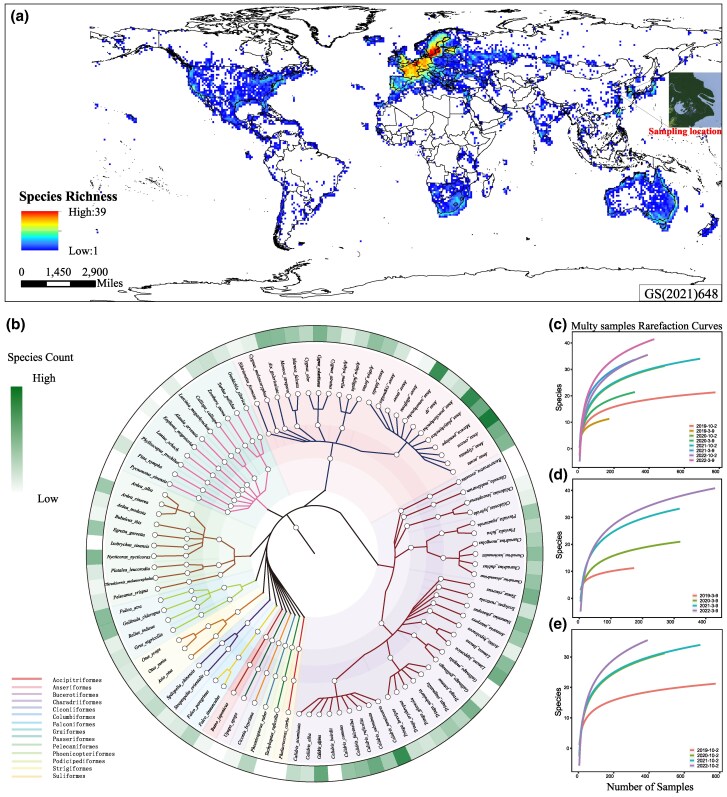
Sampling details and evaluation of sample representativeness. a) Global distribution richness of migratory bird species sampled in Shanghai. Each point on the map represents a location where birds have been observed, with the data visualization indicating the number of species diversity. The gradient scale from low to high values signifies increasing species richness. b) The taxonomic tree of species sampled in this study. The central point represents Aves, branching out to the orders, families, genera, and species names sampled. Lines of different colors represent different orders. The logarithmic values of the number of samples for species marked on the outer circle are represented by a heatmap. The heatmap intensity transitions from light to dark shades, indicating an increase in the number of species sampled. c) Rarefaction curves for species diversity sampled at different stages, months, and years within this study, d) for the sampling stages from March to September across various years, and e) for the stages from October to February across different years. The *x*-axis represents the number of samples, while the y-axis indicates the number of species sampled. Lines of different patterns indicate distinct sampling stages.

Shanghai, a significant stopover point situated at the estuary of the Yangtze River in eastern China, is on the migratory route of birds. This island is part of the East Asia-Australia migration route, which stands as one of the most important avian migration paths in the world (Olson et al. 2023). Out of the 71 globally known species of migratory birds carrying CoVs, 44 species stop over at Shanghai, accounting for 62.0% of the total (supplementary table S1, Supplementary Material online and supplementary fig. S1, Supplementary Material online). Notably, 17 of these species were identified for the first time as carriers of CoVs through this research. Distribution maps based on bird observation data show that the activity range of these birds spans globally, with primary migration destinations including Europe, North America, South Africa, and Australia (supplementary fig. S2, Supplementary Material online). The analysis also reveals that migratory birds carrying CoVs that stop over at Shanghai share similar distribution characteristics with those carrying the virus on a global scale (Fig. 1a).

From April 2019 to April 2023, a total of 5,263 samples from migratory birds were collected, including 3,305 swab samples, 1,803 fecal samples, and 154 tissue samples, covering 95 species of migratory birds across 14 orders, 28 families, and 48 genera (supplementary table S2, Supplementary Material online). The samples primarily originated from Anseriformes (*n =* 2084), Charadriiformes (*n =* 1204), and Pelecaniformes (*n =* 117), with a significant number of samples from species such as common teals (*Anas crecca*, *n =* 804), mallards (*Anas platyrhynchos*, *n =* 415), spot-billed ducks (*Anas zonorhyncha*, *n =* 344), Eurasian wigeons (*Mareca penelope*, *n =* 101), great knots (*Calidris tenuirostris*, *n =* 365), dunlins (*Calidris alpina*, *n =* 114), terek sandpipers (*Xenus cinereus*, *n =* 66), and common redshanks (*Tringa tetanus*, *n =* 62) (supplementary table S2, Supplementary Material online). Due to the seasonal migration characteristics of migratory birds, there was a considerable variation in the number and types of bird samples collected in different months. Statistical analysis of samples collected in various months showed that samples were mainly collected from August to April of the following year, with sampling peaks primarily occurring in April and November (supplementary fig. S3a, Supplementary Material online). Principal Component Analysis (PCA) results revealed significant differences in the host types of migratory birds stopping over at Shanghai between October and February of the following year and between March and September annually (supplementary fig. S3b and S3c, Supplementary Material online). Specifically, samples from October to February predominantly came from the Anatidae and Rallidae families, mainly including common teals, mallards, Eurasian wigeons, and common moorhens (*Gallinula chloropus*). In contrast, samples from March to September were mainly from the Scolopacidae and Charadriidae families, including great knots, dunlins, red-necked stints (*Calidris ruficollis*), bar-tailed godwits (*Limosa lapponica*), greater sand plovers (*Charadrius leschenaultia*), kentish plovers (*Charadrius alexandrinus*), and grey plovers (*Pluvialis squatarola*). The differences in social behavior, migration patterns, and ecological niches among these bird groups may influence the pathways and speed of virus spread, as well as the expansion of the virus–host range (Fig. 1b).

To assess the representativeness of our collected samples in reflecting the actual species richness of migratory birds, a rarefaction analysis was undertaken. Given the seasonal migration patterns of these birds, we categorized the samples gathered from April 2019 to April 2023 into eight distinct groups according to the collection period, and a thorough rarefaction analysis was performed for each group (Fig. 1c). The findings indicated a trend toward a decelerating increase in species richness, signifying that the addition of new species decreased as more samples were analyzed, approaching a saturation point that suggests nearing a complete census of the ecosystem's species diversity. However, the variation in the number of samples across different groups led to less apparent signs of this slowing trend in the rarefaction curves for the samples collected from March to September annually. Consequently, separate rarefaction analyses for the periods of October to February and March to September each year were conducted, both demonstrating a marked decline in the rate of species richness increase (Fig. 1d and 1e). This rarefaction analysis established that during the seasonal sampling, approximately 400 samples were sufficient to document between 70% and 95% of the migratory bird species visiting Shanghai from October to February annually, whereas around 200 samples could capture 80% to 90% of the species from March to September. Moreover, the rarefaction curves underscored noticeable yearly variations in the species count of birds stopping over at Shanghai during the same seasonal intervals, reflecting the dynamic fluctuations in avian species presence and potentially indicating changes in migration trajectories or habitat preferences over different years.

### Identification of CoVs in Migratory Birds

**Fig. 2.**
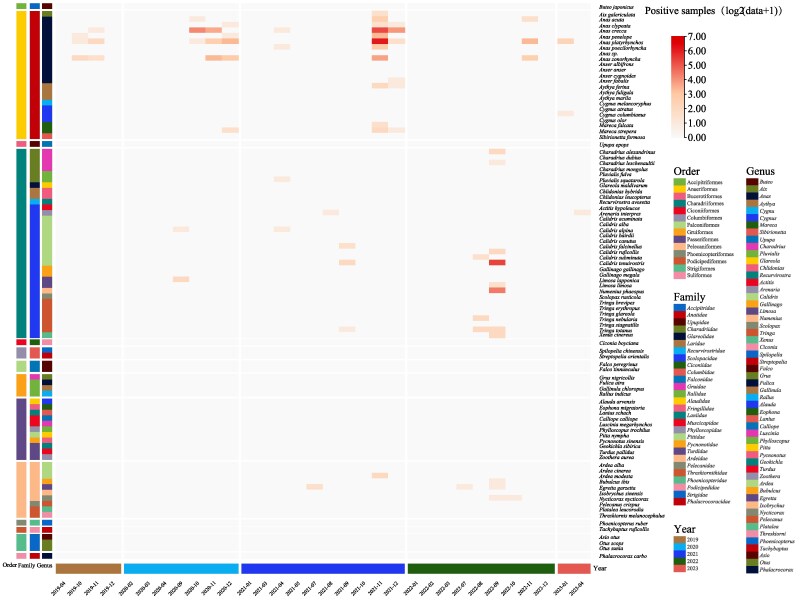
CoV detection overview. Heatmap of the number of CoV detections across different species and times. The horizontal axis represents the different months of each year, while the vertical axis represents different species. The color of the heatmap transitions from light to dark to indicate an increasing number of CoV detections. On the left side of the heatmap, a color bar annotates the genus, family, and order information for the different species. At the bottom of the heatmap, a color strip marks the different years.

In order to minimize the risk of missing coronavirus strains in the samples, this study utilized two sets of screening primers specifically designed for CoVs, conducting an in-depth investigation of 5,263 samples from migratory birds. A total of 372 samples were found to be CoV-positive, representing 7.0% of the total samples (supplementary table S2 and S3, Supplementary Material online). From these positive samples, 317 partial RNA-dependent RNA polymerase (RdRp, ∼440 bp) sequences were successfully obtained, reflecting challenges such as low viral load or sample degradation in the remaining cases. These positive samples were primarily distributed across three orders: Anseriformes (*n =* 265), Charadriiformes (*n =* 97), and Pelecaniformes (*n =* 10), encompassing four families, 17 genera, and 32 species of birds. Notably, the Anseriformes such as common teals (*n =* 81), mallards (*n =* 101), spot-billed duck (*n =* 37), and Eurasian wigeons (*n =* 11); the Charadriiformes like great knots (*n =* 43) and whimbrels (*Numenius phaeopus*, *n =* 20); and the Pelecaniformes such as little egrets (*Egretta garzetta*, *n =* 4) had a higher number of positive samples (Fig. 2 and supplementary table S3, Supplementary Material online). It is noteworthy that the detection of positive samples exhibited distinct seasonal characteristics, especially from September to December each year during the autumn and winter seasons, where the detection rate of CoVs was significantly higher than in other months, despite larger sampling volumes in some months like April 2019, February to April 2020, April to May 2021, and August 2022, but with relatively fewer positive samples (supplementary fig. S4, Supplementary Material online). Moreover, the positive rates of CoVs across different species revealed that the majority of migratory birds had positive rates ranging between 1.6% and 24.3%, for instance, common teals at 10.1% (95% CI: 8.2% to 12.3%), mallards at 24.3% (95% CI: 20.5% to 28.7%), and great knots at 11.8% (95% CI: 8.9% to 15.5%). However, it should be noted that some species such as spot-billed ducks and Eastern great egrets exhibited a positive rate of 100% (95% CI: 64.6% to 100% and 43.9% to 100% respectively), but due to the small sample sizes, these high positivity rates may not fully reflect the actual situation. (supplementary fig. S4, Supplementary Material online and supplementary table S4, Supplementary Material online).

In this study, a preliminary classification was conducted by performing sequence alignment using the 317 partial RdRp (440 bp) derived from the positive samples. The sequence alignment indicated that the identified CoV samples belonged entirely to the DeltaCoV and GammaCoV genera. Specifically, the newly identified virus strains were assigned into the subgenera *Andecovirus* (*n =* 22), *Buldecovirus* (*n =* 32), and *Herdecovirus* (*n =* 11) of DeltaCoV, as well as the subgenera *Brangacovirus* (*n =* 2) and *Igacovirus* (*n =* 250) of GammaCoV, with no viruses detected from the subgenus *Cegacovirus* (supplementary table S5, Supplementary Material online). Notably, the study identified at least two unknown evolutionary lineages, suggesting the need for whole-genome sequence analysis of these viruses for a deeper understanding of their genetic characteristics and evolutionary relationships. It is noteworthy that co-infections were detected in 31 samples. Among these, 29 samples exhibited co-infections involving two different virus species, while two samples contained co-infections involving three distinct sequences (supplementary table S5, Supplementary Material online).

**Fig. 3.**
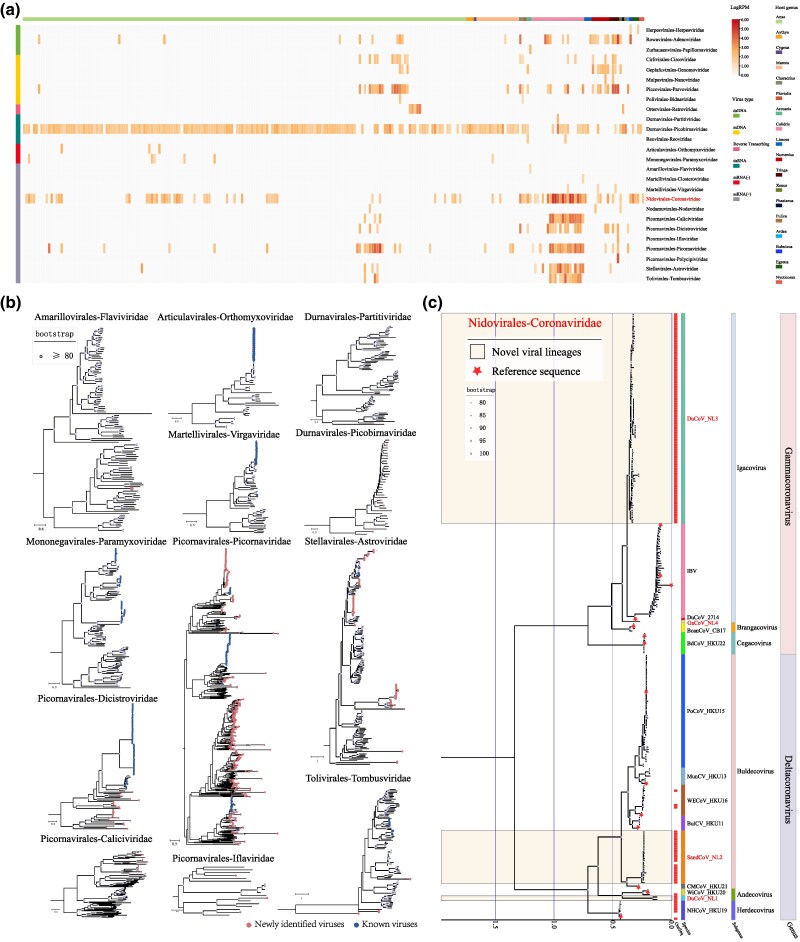
Virome diversity in CoV-Positive samples and classification of CoV. a) Viral diversity heatmap. This heatmap displays the diversity of viral families detected in coronavirus-positive samples, plotted against each sample on the horizontal axis. Each column corresponds to a sample, with host genus information annotated above. The vertical axis lists different viral families, and the number of reads per million identified for each viral family in a sample is transformed into a logarithmic scale for visualization. The intensity of colors in the heatmap indicates the relative abundance of each viral family across the samples. b) Phylogenetic tree of RNA viruses.This phylogenetic tree depicts the evolutionary relationships among RNA viruses identified in the study. Newly identified viral sequences are marked with red solid circles, while previously known viral sequences detected in this study are indicated with blue circles. This visualization helps illustrate the phylogenetic placement of new and known viruses within the broader context of RNA virus diversity. c) Phylogenetic evolutionary tree based on five conserved Regions of the GammaCoV and DeltaCoV. Reference sequences are marked with asterisks. The innermost red blocks indicate the strains identified in this study, followed by color blocks indicating the virus species, virus subgenus, and virus genus to which each virus belongs. Novel viral lineages exclusive to this study are highlighted with a light yellow shade, while novel viral lineages identified in other studies are highlighted with a light blue shade.

To efficiently obtain the whole-genome sequences of the newly identified CoVs, high-throughput sequencing was conducted. The sequencing generated approximately 4,331 GB of clean data, a total of 12,591,633,958 reads. Among these reads, 138,981,148 were identified as virus-related, with 47,492,895 being CoV-related. Additional reads associated with other virus families, such as Paramyxoviridae, Orthomyxoviridae, and Picornaviridae, were also detected. Notably, Picobirnaviridae presented the highest detection rate, while the most novel viruses were identified within the family Picornaviridae (Fig. 3a, 3b and supplementary table S6, Supplementary Material online). Combining the results from the RdRp-based classification and the CoV-related reads obtained from high-throughput sequencing, 131 representative strains were selected for whole-genome amplification. Ultimately, 120 complete CoV genome sequences were obtained (supplementary table S5, Supplementary Material online).

### Classification of Migratory Bird-Associated *Deltacoronavirus* and *Gammacoronavirus* on a Global Scale

To maximize our understanding for the diversity and ecological characteristics of all known avian-associated CoVs, this study collected publicly available virus sequences within GammaCoVs and DeltaCoVs. In addition to viral sequences, relevant meta-information concerning the samples, such as host species, sampling time/location and related literature, underwent manual curation as lineated in the “Materials and Methods” section. All the information was organized according to our predesigned generic data schema, previously employed in the ZOVER databases (Zhou et al. 2022), and stored in a local MySQL system to form the background datasets (http://www.mgc.ac.cn/cgi-bin/ZOVER/aCoV/main.cgi) (supplementary fig. S5, Supplementary Material online). As of November 1, 2024, the database includes 19,170 sequences identified from >140 animal species in approximately 100 counties globally, covering 1,162 complete sequences and 18,008 partial sequences.

Based on the latest CoV classification standards set by the International Committee on Taxonomy of Viruses (ICTV), all known 1,183 complete/partial sequences that contain five conserved domains (3CLpro, NiRAN, RdRP, ZBD and HEL1) in the ORF1ab region were first identified (Fig. 3c). Compared with public dataset, the phylogenies revealed that the 120 CoV whole-genome sequences obtained here encompass seven coronavirus species, including three newly identified species named Deltacoronavirus_anatis (DuCoV_NL1), Deltacoronavirus_Sandpipers (SandCoV_NL2), and Gammacoronavirus_Anatiscopes (DuCoV_NL3) (supplementary table S7, Supplementary Material online). Additionally, from sequences sourced from chickens in public databases (MK778364 and MK778365), a misclassified virus species initially placed under IBV was identified and was named Gammacoronavirus_Novel-IBV (GaCoV_NL4). It is particularly noteworthy that DuCoV_NL3, initially thought to belong to *Igacovirus* under DuCoV_2714 and first found in domestic ducks, was later extensively detected in migratory birds from the orders Anseriformes and Charadriiformes. To date, only one whole-genome sequence (NC_048214) of this virus species is available in public databases, yet this study achieved a breakthrough by obtaining 87 complete genome sequences related to DuCoV_2714. However, strict classification criteria indicated that DuCoV_2714 identified in domestic ducks (NC_048214) and the strains identified in migratory birds should be classified into two distinct species. Despite the close phylogenetic relationship in the RdRp region between DuCoV_NL3 and DuCoV_2714, the genomic differences at the whole-genome level are significant. The circulating strains of DuCoV_2714 in migratory birds are likely all related to DuCoV_NL3, whereas DuCoV_2714 may result from cross-species transmission. Subsequent genomic structure and evolutionary analysis further revealed the differences between DuCoV_NL3 and DuCoV_2714.

### Evolutionary History of Migratory Bird-Associated *Deltacoronavirus* and *Gammacoronavirus*

**Fig. 4.**
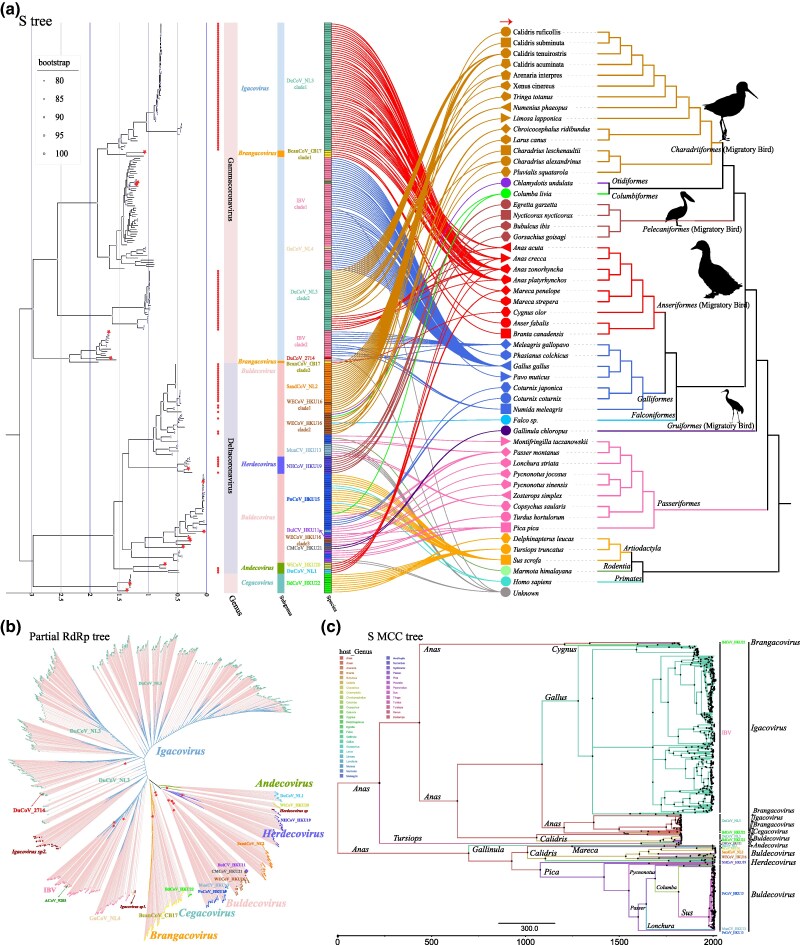
Phylogenetic tree analysis. a) Phylogenetic tree and host species phylogenetic tree based on the S protein region and cytb, respectively. On the left is the phylogenetic tree constructed based on S protein, with red circles marking the strains identified in this study and different color blocks indicating the corresponding genus, subgenus, and species. On the right is the host species phylogenetic tree based on cytb sequences of various species, with hosts of the same order marked in the same color but different shapes. Moreover, the hosts corresponding to the virus strains on the left are connected to their respective species with lines colored consistently with the host's order. b) Phylogenetic tree based on partial RdRp. Names of various virus species are annotated on the phylogenetic tree. c) Maximum clade credibility (MCC) tree constructed using BEAST based on the S sequences. The species and genus to which the virus strains belong are annotated on the right. Hosts at different genus levels are indicated in different colors, with the color of the Phylogenetic tree branches matching the color of the hosts, and hosts are marked on the branches. Additionally, the size of the nodes on the phylogenetic tree is positively correlated with the host probabilities.

To delve into the evolutionary history of CoVs carried by migratory birds, this study initially extracted all available full-length and partial RdRp, and S protein sequences from our database. After 95% de-duplicating IBV and PDCoV sequences, the IQ-TREE 2 software was used, employing the maximum likelihood method, to construct phylogenetic trees. These analyses aimed to unveil the genetic diversity of CoVs within migratory birds and their evolutionary relationships within the GammaCoV and DeltaCoV (Fig. 4a, 4b and supplementary fig. S6, Supplementary Material online). Phylogenetic analysis based on the RdRp indicated that among the 16 classified CoV species within the DeltaCoV and GammaCoV genera, nine CoV species have a migratory bird as original host. These include the DuCoV_NL3 and DuCoV_2714 of the subgenus *Igacovirus*, BcanCoV_CB17 of the *Brangacovirus*, DuCoV_NL1 and WiCoV_HKU20 of the *Andecovirus*, SandCoV_NL2, WECoV_HKU16, and CMCoV_HKU21 of the *Buldecovirus*, and NHCoV_HKU19 of the *Herdecovirus*. Analysis based on the S protein phylogenetic tree revealed complex genetic evolutionary relationships between CoVs carried by migratory birds and those from non-migratory birds, including the IBV and GaCoV_NL4 of the *Igacovirus*, and PoCoV_HKU15 and BulCV_HKU11 of the *Buldecovirus* in the S protein tree. Furthermore, the phylogenetic tree based on partial RdRp showed two clusters of unclassified viruses within the *Igacovirus*, one cluster from black-headed gulls (*Chroicocephalus ridibundus*) of Charadriiformes, named Igacovirus sp1, and another primarily from rock pigeons (*Columba livia*) of Columbiformes, named Igacovirus sp2. Additionally, a cluster of unclassified viruses from the black-faced spoonbills (*Platalea minor*) of Pelecaniformes was identified within the subgenus *Herdecovirus* and named Herdecovirus sp (Fig. 4b and supplementary table S5, Supplementary Material online).

In GammaCoV, this study obtained 87 complete genome sequences of CoVs belonging to DuCoV_NL3 of *Igacovirus*, with whole-genome sequence homology ranging from 69.26% to 100% among them (supplementary table S5 and S8, Supplementary Material online). The closest known CoV related to DuCoV_NL3 is DuCoV_2714 (NC_048214), with the highest whole-genome sequence homology of 75.24% (supplementary table S8, Supplementary Material online). This newly classified CoV species, DuCoV_NL3, along with DuCoV_2714, exhibits a complex genetic evolutionary relationship, primarily hosting migratory birds from the orders Anseriformes and Charadriiformes. Phylogenetic analysis based on the RdRp revealed that DuCoV_NL3 related CoVs and DuCoV_2714 form a closely related evolutionary cluster in RdRp, sharing 94.46% to 97.49% sequence homology (supplementary fig. S6, Supplementary Material online and supplementary table S9, Supplementary Material online). However, analysis of the S protein tree revealed significant genetic differentiation in DuCoV_NL3 related CoVs based on host diversity, resulting in two distinct clustering groups, DuCoV_NL3 clade1 and clade2. CoVs in DuCoV_NL3 clade1 primarily originate from migratory birds within the order Anseriformes, such as northern pintails (*Anas acuta*), common teal, spot-billed ducks, and mallards. Meanwhile, DuCoV_NL3 clade2 is divided into Anseriformes and Charadriiformes clusters based on different hosts, with representative hosts including spot-billed ducks and great knots. Notably, IBV-related CoVs also formed two evolutionary clusters with host differences in the S protein tree, IBV clade1 and clade2. IBV clade1 is more closely related to both DuCoV_NL3 clade1 and clade2, while IBV clade2 clusters with DuCoV_2714, sharing 58.31% to 69.01% sequence homology (Fig. 4a and supplementary table S10, Supplementary Material online). Although strains related to BcanCoV_CB17 were detected in bean geese (*Anser fabalis*) and tundra swans (*Cygnus columbianus*), whole-genome sequences were not obtained. BcanCoV_CB17 strains, primarily found in Canada geese (*Branta canadensis*), bean geese (*Anser fabalis*), grey geese (*Anser anser*) and Mute swans (*Cygnus olor*), are divided into two evolutionary clusters in the S protein, BcanCoV_CB17 clade1 and clade2. The former clusters with DuCoV_NL3 clade1, sharing 50.57% to 61.68% sequence homology, while the latter clusters with IBV clade2, sharing 53.02% to 56.73% sequence homology (supplementary table S10, Supplementary Material online). Moreover, the newly classified GaCoV_NL4 related CoVs from domestic fowls (*Gallus gallus*), while forming an independent branch closer to DuCoV_NL3 in RdRp, cluster with IBV clade1 in the S protein, sharing 60% to 95.38% sequence homology (Fig. 4a, supplementary fig. S6, Supplementary Material online and supplementary table S10, Supplementary Material online).

In DeltaCoV, this study obtained three whole-genome sequences of CoVs from the *Andecovirus*, two of which belong to a newly classified CoV species, DuCoV_NL1, with a whole-genome sequence homology of 78.36% (Fig. 4a, supplementary fig. S6, Supplementary Material online and supplementary table S5 and S8, Supplementary Material online). One sequence belongs to WiCoV_HKU20, with a sequence homology of 76.69% to known viruses of this species (supplementary table S5 and S8, Supplementary Material online). CoVs in the *Andecovirus*, primarily originating from Anseriformes migratory birds such as spot-billed ducks and common teals for DuCoV_NL1 and Eurasian wigeons for WiCoV_HKU20, showed no significant changes in evolutionary clustering in the RdRp and S protein. In *Buldecovirus*, 24 whole-genome sequences of CoVs were obtained, 21 of which belong to the newly classified SandCoV_NL2, and three to WECoV_HKU16 (supplementary table S5, Supplementary Material online). CoVs of SandCoV_NL2, mainly from great knots and ruddy turnstones (*Arenaria interpres*) of Charadriiformes, shared a whole-genome sequence homology of 75.43% to 100% and displayed stable classification trends in the RdRp and S protein trees (Fig. 4a, supplementary fig. S6, Supplementary Material online and supplementary table S8, Supplementary Material online). WECoV_HKU16 related CoVs differentiated into three evolutionary clusters with host differences in the S protein tree, WECoV_HKU16 clade1, clade2, and clade3. WECoV_HKU16 clade1 strains from sharp-tailed sandpipers (*Calidris acuminata*) clustered with SandCoV_NL2, sharing 71.76% to 98.18% sequence homology (supplementary table S9, Supplementary Material online). WECoV_HKU16 clade2 exhibited host diversity, including migratory birds from Charadriiformes and non-migratory birds from Falconiformes and Otidiformes. WECoV_HKU16 clade3’s hosts are mainly from Swinhoe's white-eyes (*Zosterops simplex*) of Passeriformes, clustering with a strain from Eurasian magpies (*Pica pica*) (MW349841) in PoCoV_HKU15, sharing 71.94% sequence homology (supplementary table S10, Supplementary Material online). No strains related to CMCoV_HKU21, detected in common moorhens and previously found only by a research team in Hong Kong, were found. Its sequence is closest to SandCoV_NL2 in the RdRp tree, with a sequence homology of 85.47% to 86.01%, but it shows a closer relationship with non-migratory bird strains from Passeriformes in the S protein tree, including BulCV_HKU11 and some strains in PoCoV_HKU15. In *Herdecovirus*, six whole-genome sequences were obtained, all belonging to NHCoV_HKU19, with hosts primarily from Pelecaniformes migratory birds such as little egrets, black-crowned night-herons (*Nycticorax nycticorax*), and Western cattle egrets (*Bubulcus ibis*). The genetic clustering of CoVs belonging to NHCoV_HKU19 appeared stable in both RdRp and S protein trees, with no divergence in clustering trends (Fig. 4a and supplementary fig. S6, Supplementary Material online).

To trace the ancestral hosts of CoVs within the GammaCoV and DeltaCoV genera and to elucidate the evolutionary diversification of these viruses over time, the complete RdRp sequence was initially employed for ancestral reconstruction. However, due to the absence of a detectable temporal signal, the S protein was subsequently utilized, given its closer association with host specificity. The analysis was conducted using S sequences with recombination events removed, employing a Bayesian ancestral reconstruction approach (Fig. 4c and supplementary table S5, Supplementary Material online). Over time, these viruses diversified into three distinct subgenera, accompanied by corresponding diversification in host species. The analysis with the maximum clade credibilty (MCC) tree indicated that CoVs of the GammaCoV and DeltaCoV primarily originated from birds of the genus *Anas* within the order Anseriformes. Specifically, viruses of the Igacovirus subgenus first diverged from hosts in the genus Anas, with subsequent divergence leading to the emergence of the IBV virus species in the genus Gallus, suggesting that IBV also originated from Anas. *Gallus* serves solely as the host origin for the IBV species and not for the ancestral hosts of the *Igacovirus* subgenus, indicating that our ancestral host reconstruction analysis is not influenced by sample size. Furthermore, viruses of the *Buldecovirus* subgenus have transitioned from hosts in genus *Anas* to the *Pica* genus, followed by *Passer* and *Columba* genera hosts, ultimately reaching *Sus* genus hosts. This suggests that maybe some animals within the *Pica*, *Passer*, and *Columba* genera (such as magpie, sparrows and pigeons) have contact with pigs, forming a hidden transmission chain. It is noteworthy that DuCoV_2714 shares a diversification trend with DuCoV_NL3, but considering its close phylogenetic relationship with IBV in other genomic regions, it is inferred that DuCoV_2714 might have originated from multiple recombination events between DuCoV_NL3 and IBV-related CoVs during evolution.

### Recombination Analysis for Migratory Bird-Associated *Deltacoronavirus* and *Gammacoronavirus*

**Fig. 5.**
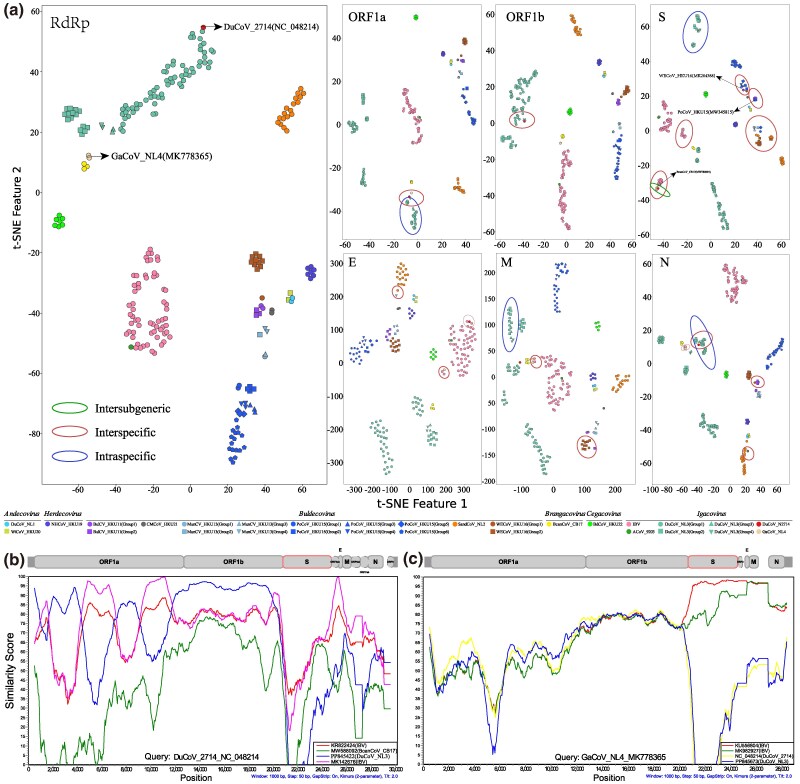
Recombination analysis. a) T-SNE clustering plot. The clustering based on sequence identity and Phylogenetic tree based on RdRp, ORF1a, ORF1b, S protein, E protein, M protein, and N protein is displayed. The same color represents the same virus species, and different shapes within the same color indicate different groups formed by this virus species on the phylogenetic tree. b) and c) Recombination analysis using Simplot. Important sequences are analyzed for recombination using Simplot, with lines of different colors representing different virus strains.

Based on the phylogenetic analyses in the previous section, potential recombination signals were detected. To clarify which recombination events occurred and which sequences were involved, recombination detection was performed with RDP5 software for whole-genome sequences. To minimize dependency among different methods, only those events identified as recombinations by all seven methods in RDP5 were considered true recombination events. Based on this criterion, the identified recombination events were further refined manually using a phylogenetic tree, which resulted in the detection of 237 recombinant sequences (supplementary table S5, Supplementary Material online). To identify recombination breakpoints in GammaCoVs and DeltaCoVs, breakpoint detection was conducted on whole-genome sequences (supplementary fig. S7, Supplementary Material online). The results suggest that recombination breakpoints tend to occur at the junctions of various gene segments. To visualize the recombination patterns within different gene segments more clearly, we applied t-SNE machine learning methods to cluster and display the recombination status across the genome (Fig. 5a and supplementary fig. S8, Supplementary Material online). To aid understanding of the genomic locations of different segments, the genomic structure of various GammaCoV and DeltaCoV subgenera is provided for reference (supplementary fig. S9, Supplementary Material online). Across multiple regions of the CoV genome, we identified anomalous clustering phenomena within different groups of the same species, between species, and even across subgenera, particularly in the S protein region. This indicates complex genetic recombination relationships among CoVs carried by migratory birds, as well as between migratory and non-migratory birds. For instance, the DuCoV_2714 strain (NC_048214) clusters with the DuCoV_NL3 Group1 strains in the RdRp region, with the DuCoV_NL3 Group4 strains in ORF1a, with the DuCoV_NL3 Group3 strains in the ORF1b, with IBV and BcanCoV_CB17 strains in the S protein, with IBV strains in E protein, and again with the DuCoV_NL3 strains in the M protein and N protein. Further analysis using Simplot revealed changes in sequence consistency across different genomic regions between DuCoV_2714 and strains of DuCoV_NL3, IBV, and BcanCoV_CB17 (Fig. 5b and 5c). The identification of DuCoV_2714 in domestic ducks, featuring genomic elements of DuCoV_NL3 from migratory birds and related strains like the domestic chicken's IBV, not only reveals the complex genetic exchange and recombination mechanisms among CoVs across hosts but also underscores the key role of migratory birds in the diversity and transmission dynamics of CoVs.

### Transmission Characteristics of Migratory Bird-Associated *Deltacoronavirus* and *Gammacoronavirus*

**Fig. 6.**
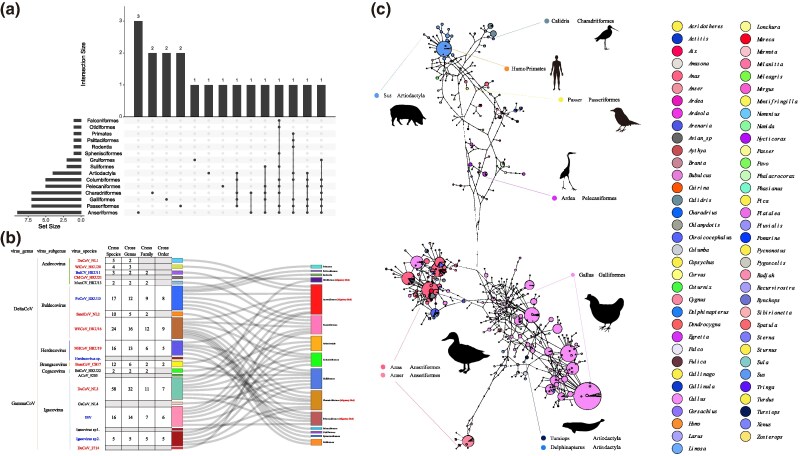
Transmission and virus–host network. a) This UpSet plot illustrates the intersection of virus species carried by hosts from different avian orders. The Set Size represents the total number of virus species associated with each host order (e.g. Anseriformes has a Set Size of 9, indicating nine virus species carried by hosts of this order). The Intersection Size represents the number of virus species shared between different host orders. Black dots below the bars indicate which host orders share virus species: single black dots without connections denote virus species unique to one host order, while connected dots indicate species shared among multiple orders. For example, Anseriformes has three virus species unique to it (shown by a single black dot), while connected dots represent intersections where virus species are shared by multiple host orders. b) Numbers of transmission events at various taxonomic levels on different levels of host. The number of transmissions at the levels of cross-species, cross genus, cross family, and cross order for different virus species are shown at the corresponding positions. Each virus species is marked with a different color block, and the species involved in that virus species are connected to the host order they belong to with lines. On the right, different hosts corresponding to different orders are indicated with different colors. c) The haplotype network illustrates the relationships among viral sequences from various host genera. Each color represents a different host genus, with similar colors used for genera within the same host order. The size of each circle is proportional to the number of viral sequences it contains. Silhouettes of the main host orders are highlighted and labeled for clarity.

To comprehensively reveal the transmission characteristics of CoVs carried by migratory birds both within and between host species, this study utilized the 317 partial RdRp (440 bp) sequences obtained from CoV-positive samples as a foundation, and integrated 2,826 sequences of CoVs belonging to the DeltaCoV and GammaCoV genera from our database. After correcting the host information from the public data, we obtained 3,215 sequences with clear host designation and 11 virus sequences with hosts identified at the genus level (supplementary table S5, Supplementary Material online). Analysis of the data revealed that most CoV species have their preferred host species, such as common teals for DuCoV_NL1 and Eurasian wigeons and Northern shovelers (*Anas clypeata*) for WiCoV_HKU20 (supplementary fig. S10, Supplementary Material online). These observations indicate that certain CoV species, including DuCoV_NL3 and IBV, exhibit a significant range of host diversity, likely due to their history of multiple cross-species transmission events. Further analysis indicated that these transmission events occurred at the levels of animal species, genera, families, and even orders. An examination of the host characteristics of 16 classified virus species within the DeltaCoV and GammaCoV genera revealed that 13 classified CoV species were found in hosts spanning more than two species and genera, 10 virus species were discovered in hosts across more than two families, and six virus species were identified in hosts from more than two orders (Fig. 6a and 6b).

After analyzing whether the transmission of CoVs is associated with migratory birds, it was found that among the 12 classified CoV species that have undergone transmission, seven of which have migratory birds as their primary hosts, and three CoV species primarily hosted by non-migratory birds and then transmitted to migratory birds (Fig. 6a and 6b). Notably, the newly identified DuCoV_NL3-related CoV in this study has been found in 58 host species, marking it as the CoV species with the broadest known range of host species, which includes both migratory and non-migratory birds. Following DuCoV_NL3-, WECoV_HKU16-related CoV has the most extensive host range at the order level, circulating among both migratory and non-migratory birds (Fig. 6b and supplementary table S5, Supplementary Material online). Additionally, NHCoV_HKU19 also exhibits circulation among both migratory and non-migratory birds.

To delve deeper into the transmission characteristics of viruses within the DeltaCoV and GammaCoV among different hosts, this study constructed host–virus networks at both the host family and host species levels (Fig. 6c). Through network analysis, we discovered that host animals within the families Anatidae, Phasianidae, and Columbidae generate the most connections among different virus species, indicating these hosts play a pivotal role in the transmission of viruses. Analyzing the unique ecological niches occupied by birds in these three families revealed that birds within Anatidae straddle the ecological niches of migratory birds and poultry, birds in Phasianidae are primarily in the poultry niche, and pigeons in Columbidae occupy an ecological niche between non-poultry domesticated birds and resident birds. Together, they form the interface of contact among migratory birds, resident birds, and poultry. Moreover, the host–virus network at the host species level revealed that viruses related to DuCoV_NL3 exhibit the most significant host diversity, highlighting their potential for transmission among different hosts. In this study, using DuCoV_2714 as an example, a pathogen transmission model of CoVs among migratory birds, resident birds, and poultry was constructed around key hosts.

**Fig. 7.**
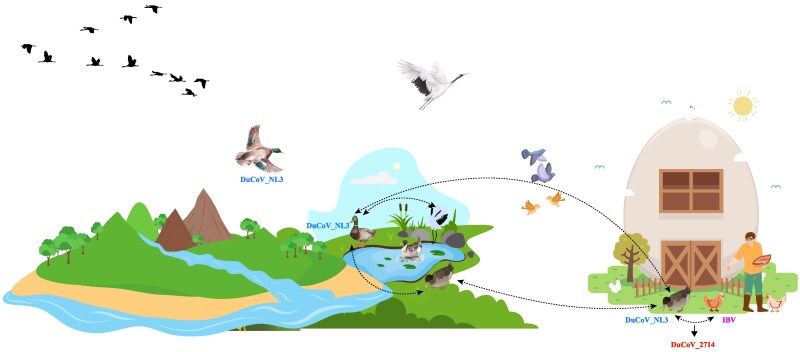
Ecological schematic diagram. This diagram illustrates the hypothesized transmission pathways of DuCoV_2714 among domestic ducks, wild ducks of the Anatidae family, and chickens. Black arrows depict the potential paths of viral spread.

DuCoV_2714, identified in domestic ducks, exhibits recombination characteristics closely related to the DuCoV_NL3 strain found in wild ducks and the IBV strain in chickens. This formation process reflects, to a certain extent, the transmission dynamics of CoVs between migratory birds and poultry. Here, the formation process of DuCoV_2714 was speculated (Fig. 7). Initially, domestic ducks carrying DuCoV_NL3 might have acquired it through contact with migratory birds of the Anatidae family, such as spot-billed ducks or mallards, or possibly domestic pigeons from the family Columbidae could have acquired it from spot-billed ducks or mallards and subsequently transmitted it to domestic ducks upon returning to the farm. Later, in mixed-species rearing environments with chickens, ducks infected with DuCoV_NL3 might have co-infected with IBV carried by chickens, leading to the recombination and formation of the DuCoV_2714 strain. Given that multiple recombination events were detected in the genome sequence of DuCoV_2714, and most recombinant regions show varying genetic distances to known strains of IBV or DuCoV_NL3, such transmission may have occurred repeatedly over an extended period. Since no strains closely related to DuCoV_2714 at the genomic level have been detected in wild ducks, it remains uncertain whether this strain has further spread among migratory birds. This necessitates enhanced monitoring of viruses carried by migratory birds to more comprehensively analyze the transmission dynamics and patterns of CoVs between migratory birds and poultry.

## Discussion

Birds, as the primary reservoir hosts for GammaCoV and DeltaCoV, carry genetically diverse CoVs, posing an undeniable potential threat to socio-economic stability and public health (Milek and Blicharz-Domanska 2018; Domanska-Blicharz, Milek-Krupa and Pikula 2021; Marchenko et al. 2022). Given that birds with seasonal migration patterns can cross national boundaries, operate globally (Somveille et al. 2013), and come into extensive contact with other birds (including migratory and non-migratory species) and poultry during their migration, it is imperative to conduct thorough investigations into the CoVs they carry (Rahman et al. 2021). This study selected Shanghai, a key stopover on the East Asia-Australia migration route, for deep sampling of migratory birds over a period of >4 years. From 5,263 samples of migratory birds, a total of 372 positive samples were identified and 120 whole-genome sequences were further obtained. These viruses belong to five genera and eight species, including three virus species that were newly identified. The research revealed the high diversity of CoVs carried by migratory birds and highlighted their critical role in the transmission of GammaCoV and DeltaCoV. These findings are crucial for understanding the potential threat posed by viruses carried by migratory birds to socio-economic stability and public health, emphasizing the necessity for enhanced surveillance. To facilitate the analysis and surveillance of avian-associated CoV sequences, this study also developed a comprehensive database equipped with a variety of visualization utilities. This platform is tailored exclusively for CoV research, offering easy browsing and analytical supports such as text query, sequence search, annotation, classification, and evolutionary analysis. With its user-friendly interface and integrated online bioinformatics tools, this database serves as a convenient and powerful tool for virologists and zoologists to conduct effective monitoring and potentially predict transmission of emerging zoonotic diseases.

Research on CoVs carried by birds faces multiple challenges, including the lack of standardized virus detection criteria (Chu et al. 2011; Chamings et al. 2018), as well as insufficient genomic sequences (Woo et al. 2009; Chen et al. 2013; Lau et al. 2018; Wille et al. 2018, 2019, 2020). These factors have limited the progress of virus classification and genetic diversity. To overcome these limitations, this study not only employed screening primers designed for the highly conserved region of the RdRp gene applicable to all known CoVs but also used screening primers specifically designed for GammaCoV and DeltaCoV. The aim was to comprehensively screen for CoV-positive samples in the collected specimens while integrating the data with public databases as much as possible. Furthermore, to efficiently obtain the genomic sequences of avian CoVs, this study conducted high-throughput sequencing on the screened positive samples. In public databases, although there are about 851 whole-genome sequences for GammaCoV and DeltaCoV, these sequences are primarily concentrated in IBV from chickens (572 sequences) and HKU15 from pigs (198 sequences). For CoVs from other birds, there are only 64 whole-genome sequences available (supplementary table S5, Supplementary Material online). This distribution clearly indicates that, apart from certain economically important animals, the understanding of the genetic diversity of CoVs carried by other birds is still very limited. Through in-depth sampling of migratory birds, this study successfully obtained 120 high-quality whole-genome sequences of CoVs, significantly enriching the genetic information of CoVs in public databases. This has irreplaceable value for a deeper understanding of the genetic diversity of CoVs carried by migratory birds and their transmission characteristics.

The types of CoVs identified in migratory birds nearly cover the entire spectrum of viruses carried by migratory birds from different regions around the world, validating the hypothesis that migratory birds contribute to the global spread of viruses(Rappole, Derrickson and Hubalek 2000; Reed et al. 2003; Feare 2010; Malik et al. 2021). Currently, GammaCoV and DeltaCoV encompass six classified subgenera, of which, apart from the subgenus *Cegacovirus* found only in marine mammals, the remaining five subgenera include CoV species with migratory birds serving as their primary natural hosts. Moreover, among the known 15 avian-related CoV species, nine are associated with migratory birds, and our identified strains cover eight of these CoV species, including three newly classified CoV species here. The only migratory bird-related virus species not identified in this study is CMCoV_HKU21, detected in *G. chloropus* of the Gruiformes, currently identified by only one strain from a research team in Hong Kong (Woo et al. 2012). Public data suggest that the hosts for CoVs related to migratory birds mainly come from migratory birds of the orders Anseriformes, Charadriiformes, and Pelecaniformes, with a very small number of strains from the order Gruiformes. Unfortunately, we did not collect samples from migratory birds of the order Gruiformes, which is likely the main reason for the absence of related strains in our findings. This finding not only highlights the significant position of Shanghai in the migratory bird network but more importantly, reveals the capacity of CoVs carried by migratory birds to transcend geographical limitations and spread extensively through bird migration on a global scale.

The viruses carried by migratory and non-migratory birds exhibit complex recombination phenomena, highlighting the high dynamics and genetic diversity of transmission both between and within migratory and non-migratory birds. In GammaCoV, CoVs of the subgenera *Igacovirus* and *Brangacovirus* primarily have bird hosts from the orders Galliformes, Anseriformes, and Charadriiformes. Initial analyses were inclined to consider birds of the order Anseriformes as the origin hosts for GammaCoV, this hypothesis was also supported by the results of Bayesian analyses. We observed numerous recombination events in the CoVs of the subgenera *Igacovirus* and *Brangacovirus*, including recombination between these two subgenera. The precondition for recombination is the co-infection of a species by two or more strains, implying frequent transmission of this genus of CoVs among migratory and non-migratory birds (especially poultry). Additionally, migratory birds of the genus *Anas* within the order Anseriformes also might be the origin hosts for DeltaCoV. Moreover, the observed recombination events mainly occurred among non-migratory resident birds, with fewer recombination events observed in CoVs carried by migratory birds. Notably, the recombination among CoVs carried by non-migratory resident birds also features interspecies recombination. In contrast, our previous study on bat CoVs identified numerous recombination events mostly occurred within the same virus species (Han et al. 2023; Wu et al. 2023). The unique recombination patterns displayed by GammaCoV and DeltaCoV reveal their complexity in transmission and genetic diversity, meriting further in-depth research.

The IBV within GammaCoV and the PDCoV within DeltaCoV have respectively been able to cause epidemics in chicken and pig populations, posing substantial threats to the livestock industry. This study did not identify any strains directly related to IBV, yet a large number of strains related to DuCoV_2714 within the same subgenus *Igacovirus* were found in wild ducks, initially discovered in Chinese domestic ducks. Surprisingly, researchers from different countries have also identified a significant number of DuCoV_2714 related strains in wild ducks, but currently, only one complete genome sequence is recorded in the public database. Analysis results based on the whole-genome sequences of the newly identified DuCoV_2714 related CoVs suggest that DuCoV_2714 and the strains identified in wild ducks should be divided into two different virus species, with the strains identified in wild ducks named DuCoV_NL3. Although DuCoV_2714 and DuCoV_NL3 share a close relationship in the RdRp region (94.46@@% to 97.49%, nt), whole-genome comparison indicates that DuCoV_2714 is likely the result of multiple recombination events with DuCoV_NL3 and IBV-related strains.

Numerous studies have demonstrated that the risk of transmission of viruses is closely linked to the phylogenetic relationships between their hosts and the extent of overlap in their geographical distributions (Streicker et al. 2010; Huang et al. 2014; Wells et al. 2018; Stephens et al. 2019). Specifically, the closer the phylogenetic relationship between species and the wider the overlap in their geographical distributions, the greater the likelihood of sharing viruses (Huang et al. 2014; Albery et al. 2020). Therefore, viruses must overcome multiple barriers and adapt to different hosts in order to achieve transmission (Parrish et al. 2008). Through detailed analysis of the host characteristics of DuCoV_2714, DuCoV_NL3, and IBV, we developed an ecological schematic diagram describing the genesis of DuCoV_2714. In this diagram, wild ducks carrying DuCoV_NL3 transmitted the virus to domestic ducks at migratory stopover points, or possibly indirectly via non-migratory birds, such as pigeons. Subsequently, these domestic ducks, coexisting with IBV-infected chickens, became co-infected, resulting in the emergence of DuCoV_2714 through multiple recombination events. The biological kinship between domestic and wild ducks played a key role in linking migratory and non-migratory birds, particularly poultry. This scenario provides a plausible explanation for the differing recombination patterns observed among GammaCoV and DeltaCoV genera in migratory versus non-migratory birds. In the absence of significant intermediate hosts, such as domestic ducks, DeltaCoV viruses primarily recombine within resident bird populations, without interactions involving migratory birds. Furthermore, the close relationship between Porcine deltaCoV and PoCoV_HKU15-related strains carried by non-migratory birds, such as *Columba livia*, offers insights into DeltaCoV transmission patterns, warranting further exploration in future research.

In summary, this study unveils the genetic diversity of CoVs within migratory birds and their potential for global spread, providing a crucial scientific foundation for understanding and addressing future potential pandemics. Future work will need to extend over a broader geographic and biodiversity range to delve deeper into the evolutionary history, host adaptability, and transmission mechanisms of CoVs, to better prevent and control the impact of these pathogens on human society and ecosystems.

### Limitations of the Study

Despite the substantial scale of this investigation, several limitations warrant consideration. First, although four years of sampling at a major migratory stopover in Shanghai provided robust data, it may not fully represent the global diversity of avian CoVs. A broader geographic range, encompassing different migratory flyways and various ecological settings, could offer a more comprehensive view of distribution and transmission dynamics of these viruses. Second, some temporal gaps in the sampling process might have overlooked short-term fluctuations in virus prevalence or seasonal shifts in host–virus interactions. Implementing continuous or more frequent sampling schedules could refine our understanding of avian CoV evolution and cross-species transmission potential. Addressing these limitations in future studies may further illuminate the ecological and epidemiological landscape of avian CoVs.

## Supplementary Material

msaf045_Supplementary_Data

## Data Availability

The raw data, and CoV sequence generated in this study have been deposited in the Sequence Read Archive (SRA) and Genome Sequence Archive (GSA) of the National Genomics Data Center, respectively, under accession codes PRJNA1113436 (https://dataview.ncbi.nlm.nih.gov/object/PRJNA1113436?reviewer=e34r4p81qo9r4n4f3bvfpv764n) and PRJCA026486 (https://ngdc.cncb.ac.cn/gsa/browse/CRA016941). The CoV sequences generated in this study have been deposited in the NCBI GenBank with accession numbers PP845379-PP845818.
