# Supplementary material for: Avian Migration-Mediated Transmission and Recombination Driving the Diversity of Gammacoronaviruses and Deltacoronaviruses
Source: Mol Biol Evol. 2025 Feb 18;42(3):msaf045. doi: 10.1093/molbev/msaf045 (PMC11886833; doi:10.1093/molbev/msaf045)
Supplement: msaf045_Supplementary_Data [file msaf045_supplementary_data.zip › Supplementary Figures 1-10.pdf]

Order	Family	Genus	Species	Type
Anseriformes	Anatidae	Aix	Aix galericulata	Migrant
		Anas	Anas acuta	Migrant
			Anas americana	Resident
			Anas bahamensis	Migrant
			Anas clypeata	Migrant
			Anas crecca	Migrant
			Anas gracilis	Resident
			Anas platyrhynchos	Migrant
			Anas poecilorhyncha	Migrant
			Anas superciliosa	Resident
			Anas zonorhyncha	Migrant
		Anser	Anser anser	Migrant
			Anser caerulescens	Migrant
			Anser cygnoides	Migrant
			Anser fabalis	Migrant
		Aythya	Aythya ferina	Migrant
			Aythya fuligula	Migrant
			Aythya marila	Migrant
		Branta	Branta bernicla	Migrant
			Branta canadensis	Migrant
		Cairina	Cairina moschata	Resident
		Cygnus	Cygnus columbianus	Migrant
			Cygnus olor	Migrant
		Dendrocygna	Dendrocygna javanica	Resident
		Mareca	Mareca falcata	Migrant
			Mareca penelope	Migrant
			Mareca strepera	Migrant
		Melanitta	Melanitta nigra	Migrant
		Mergus	Mergus merganser	Migrant
		Radjah	Radjah radjah	Resident
		Sibirionetta	Sibirionetta formosa	Migrant
		Spatula	Spatula clypeata	Migrant
			Spatula querquedula	Migrant
Pelecaniformes	Ardeidae	Ardea	Ardea cinerea	Migrant
			Ardea modesta	Resident
		Ardeola	Ardeola bacchus	Migrant
		Bubulcus	Bubulcus ibis	Migrant
		Egretta	Egretta garzetta	Migrant
			Egretta picata	Migrant
		Gorsachius	Gorsachius magnificus	Resident
		Nycticorax	Nycticorax nycticorax	Migrant
	Threskiornithidae	Platalea	Platalea minor	Migrant
Columbiformes	Columbidae	Columba	Columba livia	Resident
Passeriformes	Corvidae	Corvus	Corvus cornix	Resident
			Corvus frugilegus	Migrant
		Pica	Pica pica	Resident
	Estrildidae	Lonchura	Lonchura striata	Resident
	Muscicapidae	Copsychus	Copsychus saularis	Resident
	Passeridae	Montifringilla	Montifringilla taczanowskii	Resident
		Passer	Passer montanus	Resident
	Pycnonotidae	Pycnonotus	Pycnonotus jocosus	Resident
			Pycnonotus sinensis	Migrant
	Sturnidae	Acridotheres	Acridotheres cristatellus	Resident
		Sturnus	Sturnus vulgaris	Migrant
	Turdidae	Turdus	Turdus hortulorum	Migrant
	Zosteropidae	Zosterops	Zosterops simplex	Resident
Charadriiformes	Charadriidae	Charadrius	Charadrius alexandrinus	Migrant
			Charadrius leschenaultii	Migrant
		Pluvialis	Pluvialis squatarola	Migrant
	Laridae	Chroicocephalus	Chroicocephalus ridibundus	Resident
		Larus	Larus argentatus	Migrant
			Larus canus	Migrant
			Larus fuscus	Migrant
			Larus glaucescens	Migrant
			Larus heuglini	Resident
			Larus hyperboreus	Migrant
			Larus schistisagus	Migrant
		Rynchops	Rynchops niger	Migrant
		Sterna	Sterna hirundo	Migrant
	Recurvirostridae	Recurvirostra	Recurvirostra novaehollandiae	Resident
	Scolopacidae	Arenaria	Arenaria interpres	Migrant
		Calidris	Calidris acuminata	Migrant
			Calidris alba	Migrant
			Calidris alpina	Migrant
			Calidris falcinellus	Migrant
			Calidris ferruginea	Migrant
			Calidris fuscicollis	Migrant
			Calidris mauri	Migrant
			Calidris ptilocnemis	Migrant
			Calidris ruficollis	Migrant
			Calidris subminuta	Migrant
			Calidris tenuirostris	Migrant
		Gallinago	Gallinago gallinago	Migrant
		Limosa	Limosa lapponica	Migrant
			Limosa limosa	Migrant
		Numenius	Numenius phaeopus	Migrant
		Tringa	Tringa nebularia	Migrant
			Tringa totanus	Migrant
		Xenus	Xenus cinereus	Migrant
	Stercorariidae	pomarine	pomarine jaeger	Resident
Otidiformes	Otididae	Chlamydotis	Chlamydotis undulata	Resident
Suliformes	Phalacrocoracidae	Phalacrocorax	Phalacrocorax carbo	Migrant
			Phalacrocorax fuscicollis	Resident
	Sulidae	Sula	Sula leucogaster	Resident
Galliformes	Numididae	Numida	Numida meleagris	Resident
	Phasianidae	Meleagris	Meleagris gallopavo	Resident
		Coturnix	Coturnix coturnix	Migrant
			Coturnix japonica	Migrant
		Pavo	Pavo muticus	Resident
		Phasianus	Phasianus colchicus	Resident
Psittaciformes	Psittacidae	Amazona	Amazona vinacea	Resident
Gruiformes	Rallidae	Fulica	Fulica atra	Migrant
		Gallinula	Gallinula chloropus	Migrant
Sphenisciformes	Spheniscidae	Pygoscelis	Pygoscelis papua	Resident

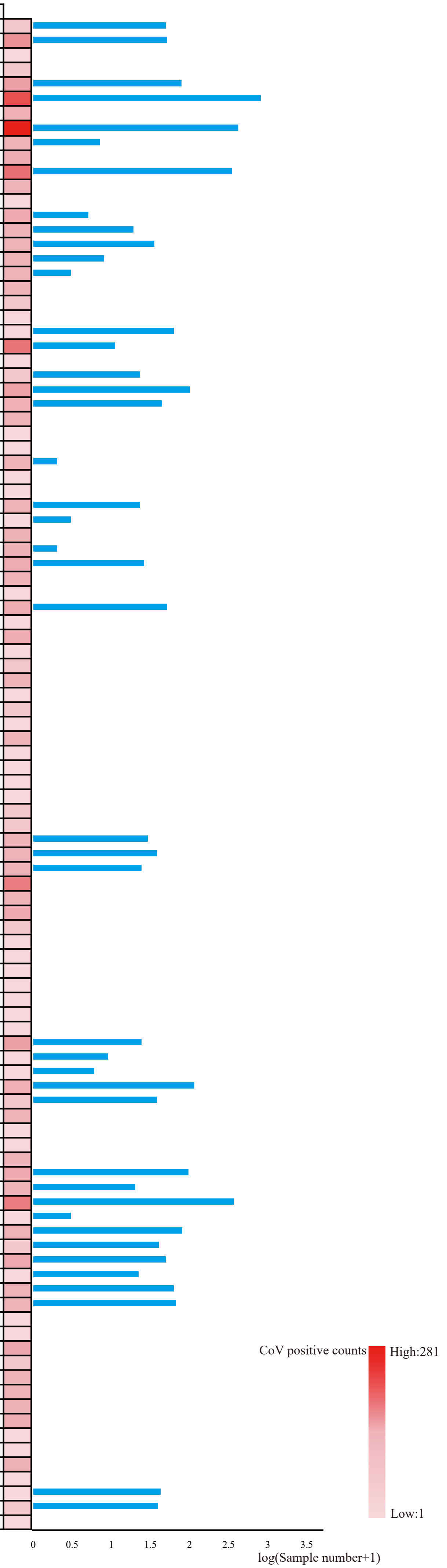

Supplementary Fig 1

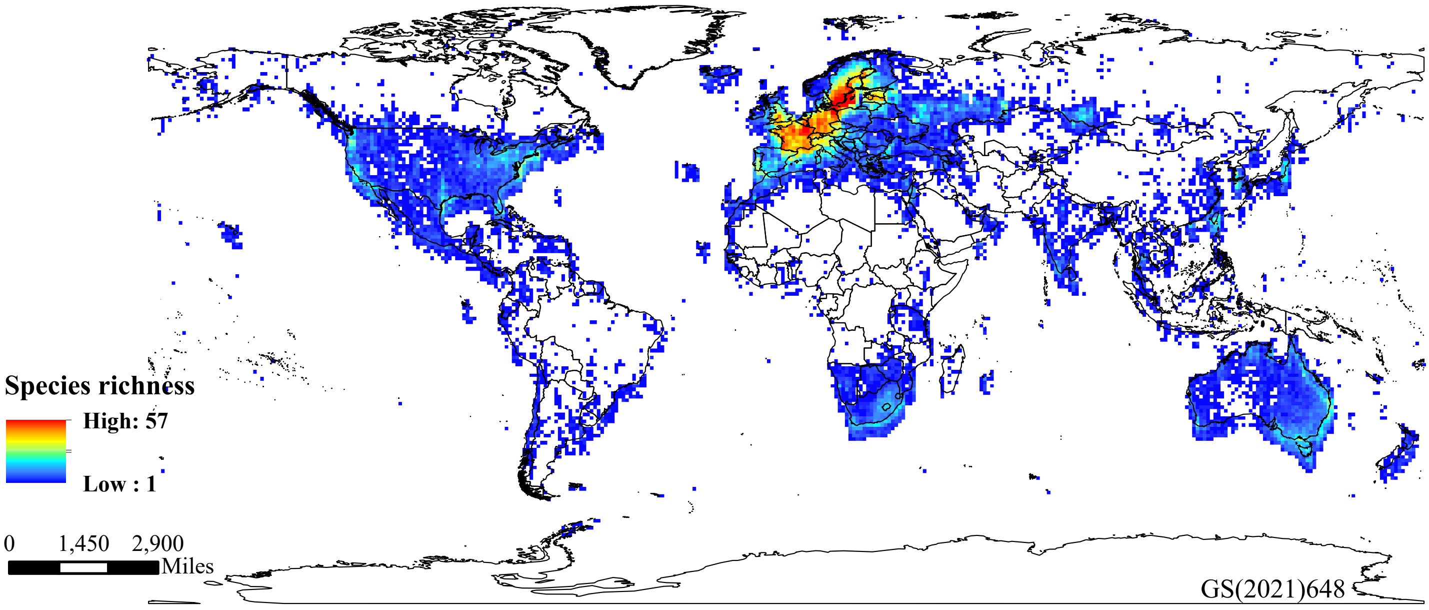

Supplementary Fig 2

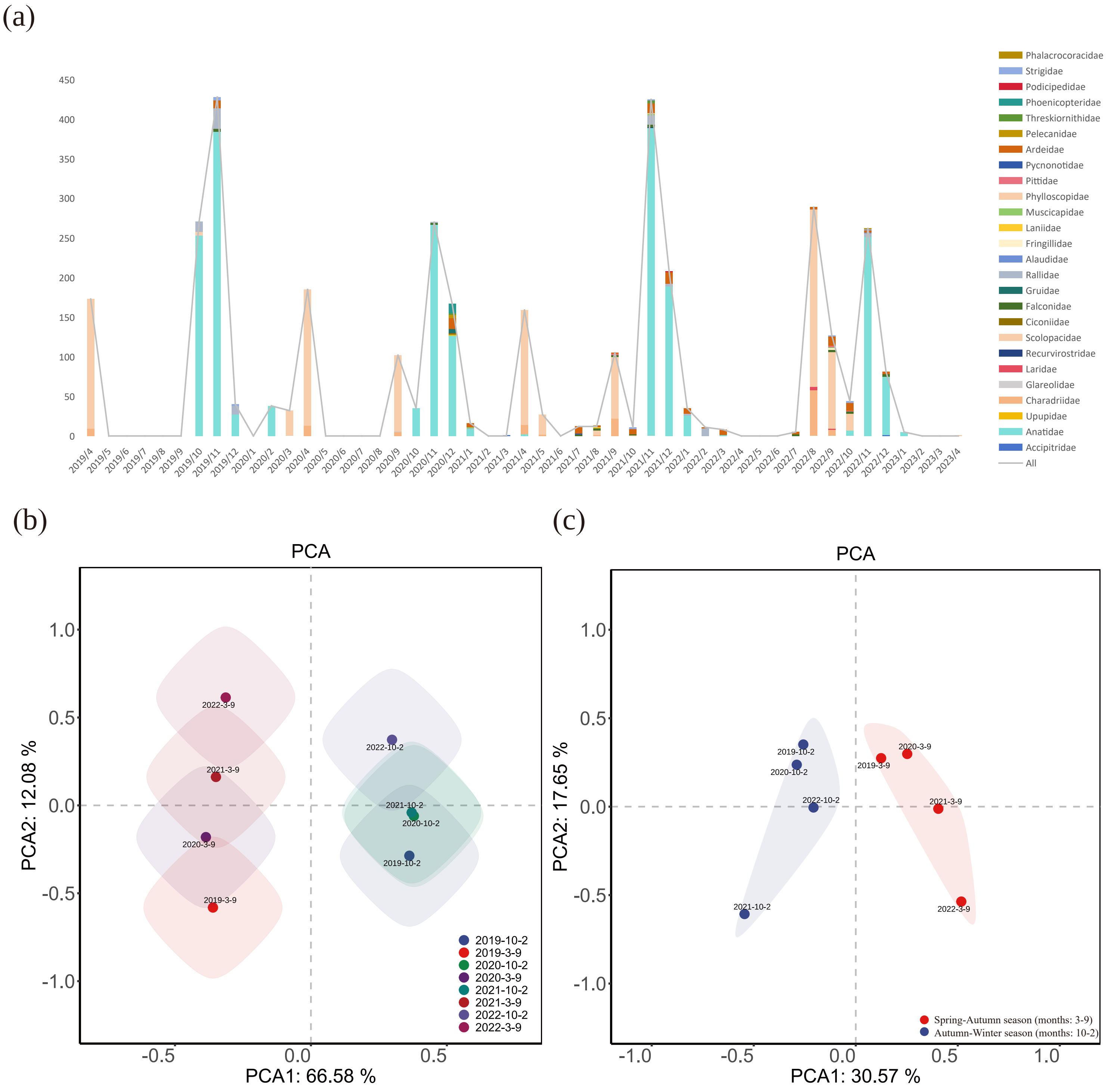

Supplementary Fig 3

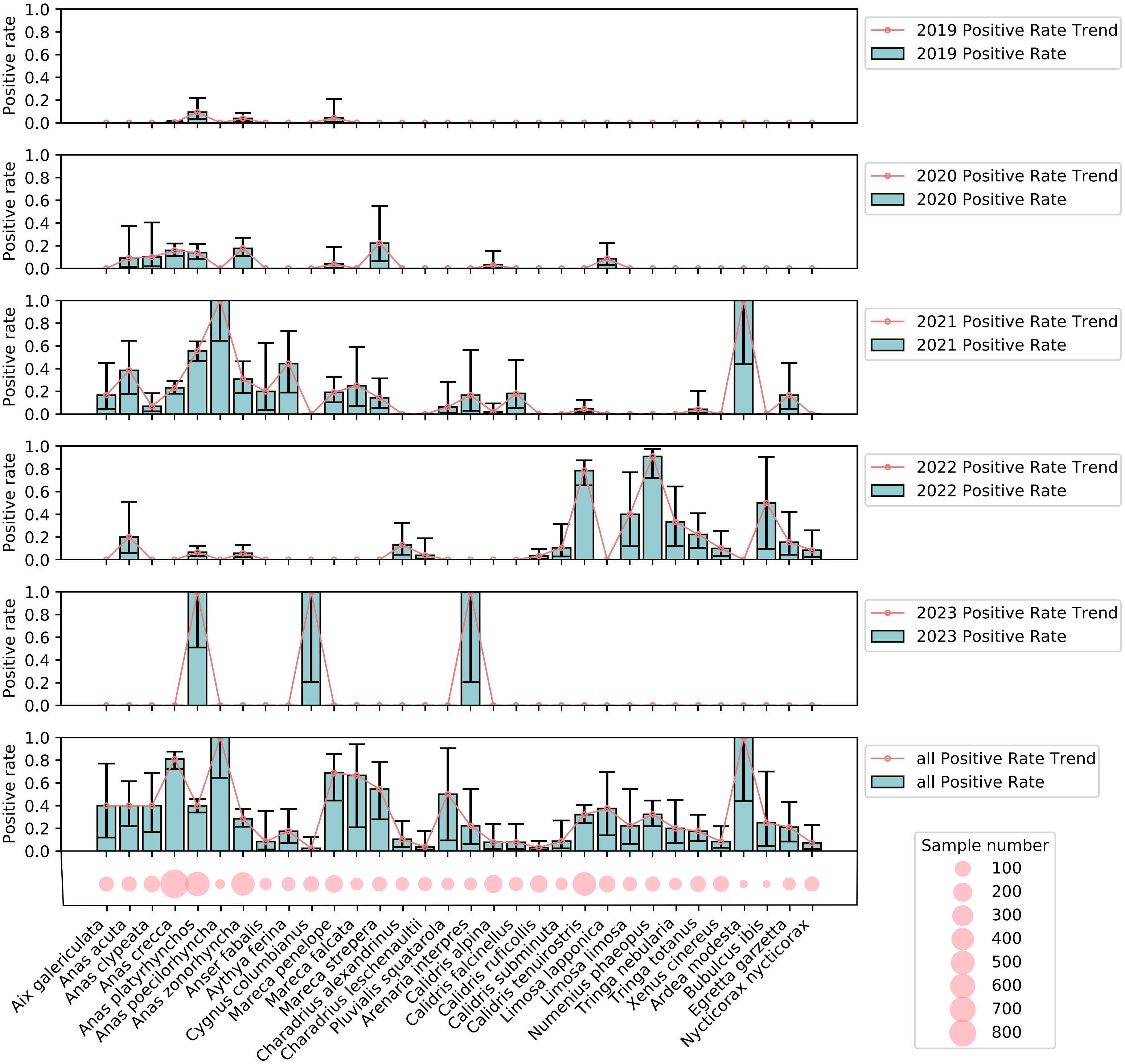

Supplementary Fig 4

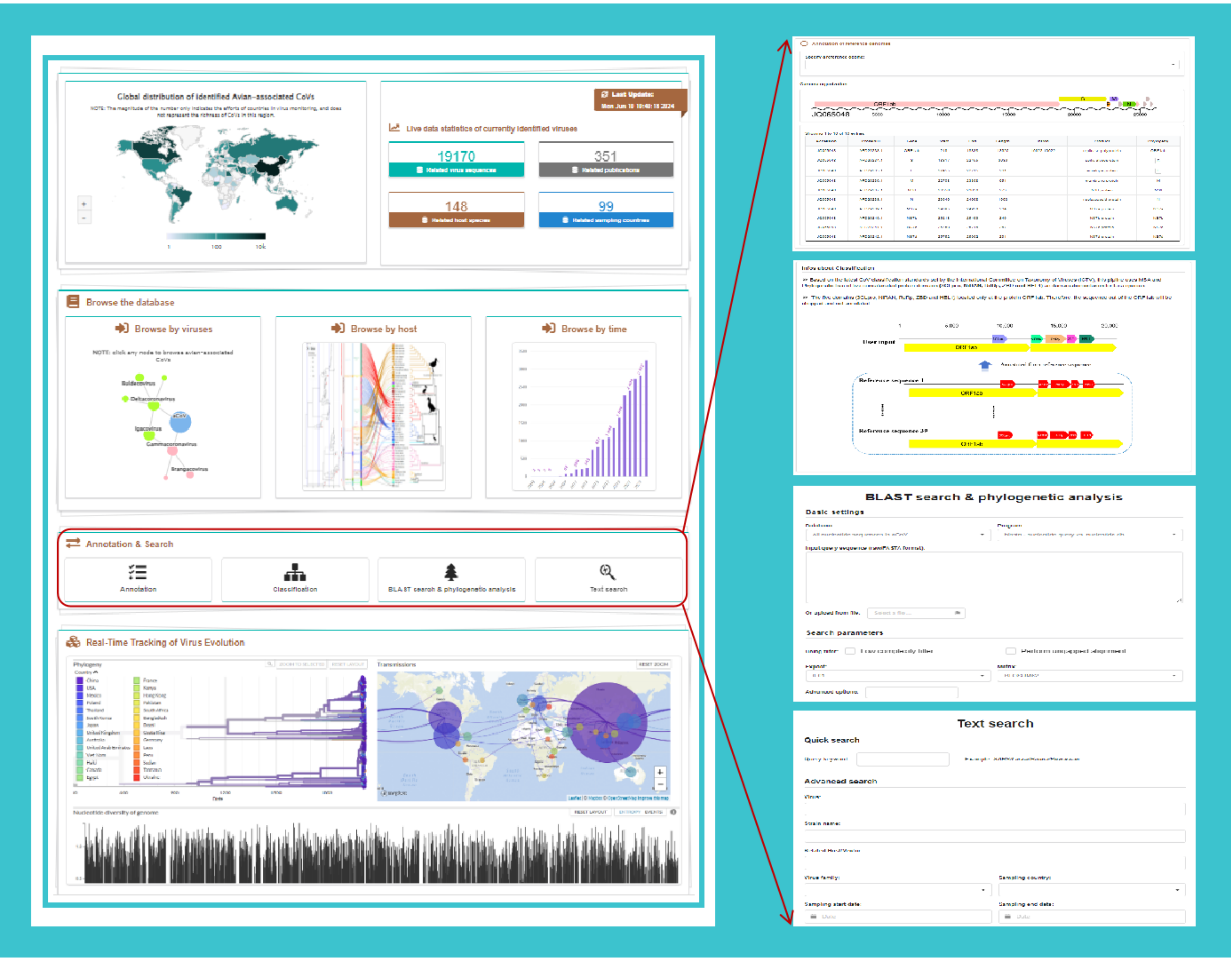

Supplementary Fig 5

RdRp tree

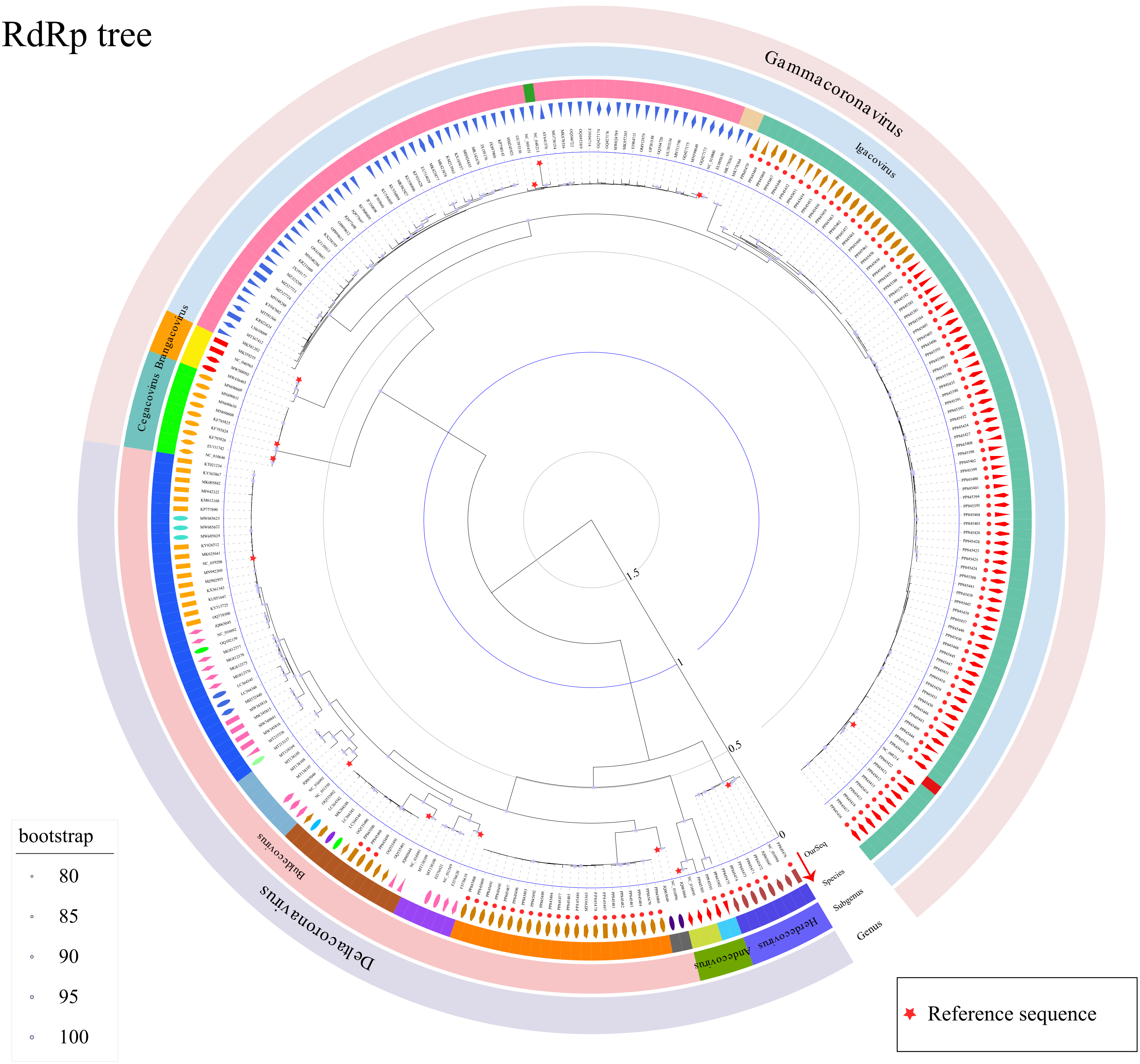

Supplementary Fig 6

Gammacoronavirus

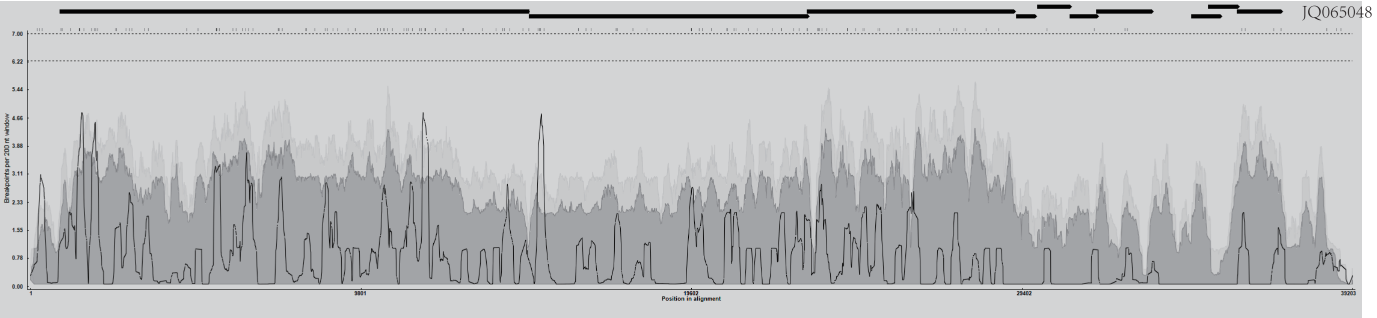

Type sequence (KF793826)

Deltacoronavirus

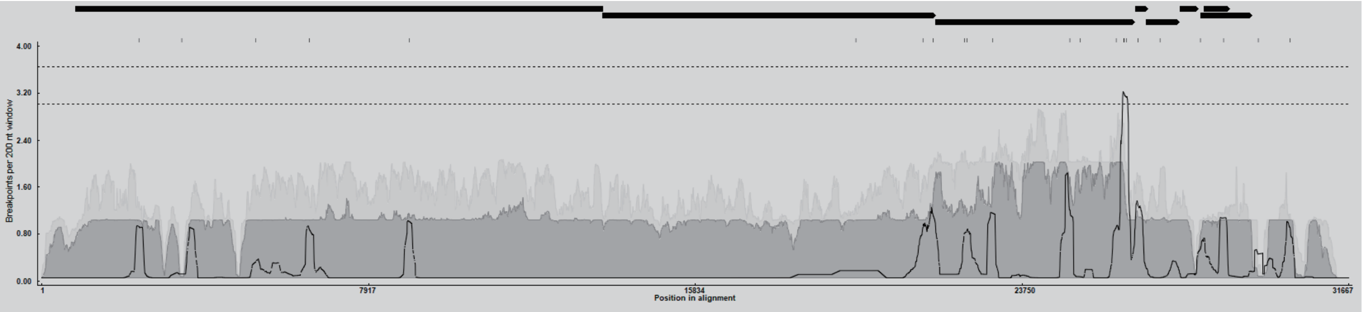

Type sequence (JQ065048)

Supplementary Fig 7

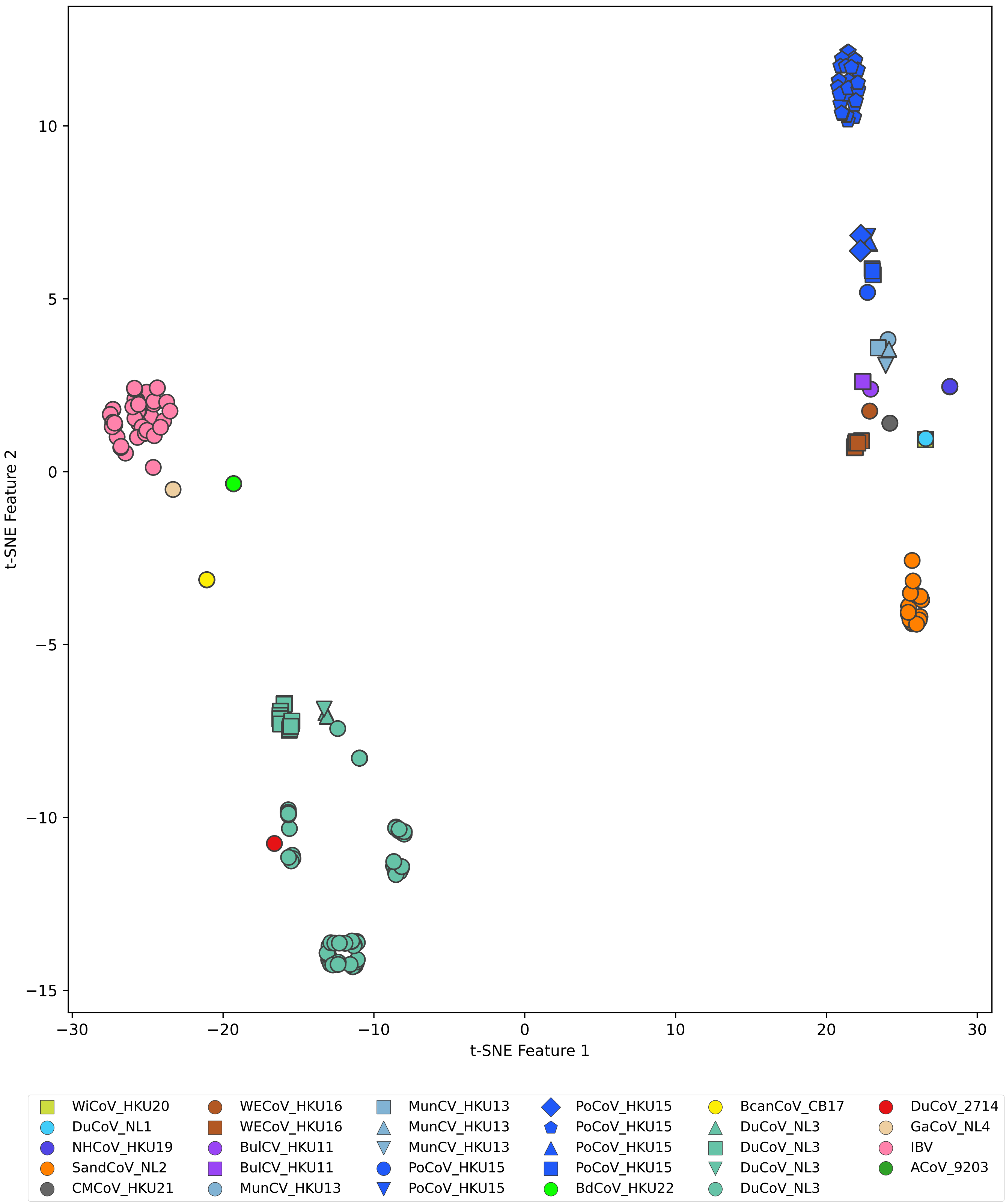

Supplementary Fig 8

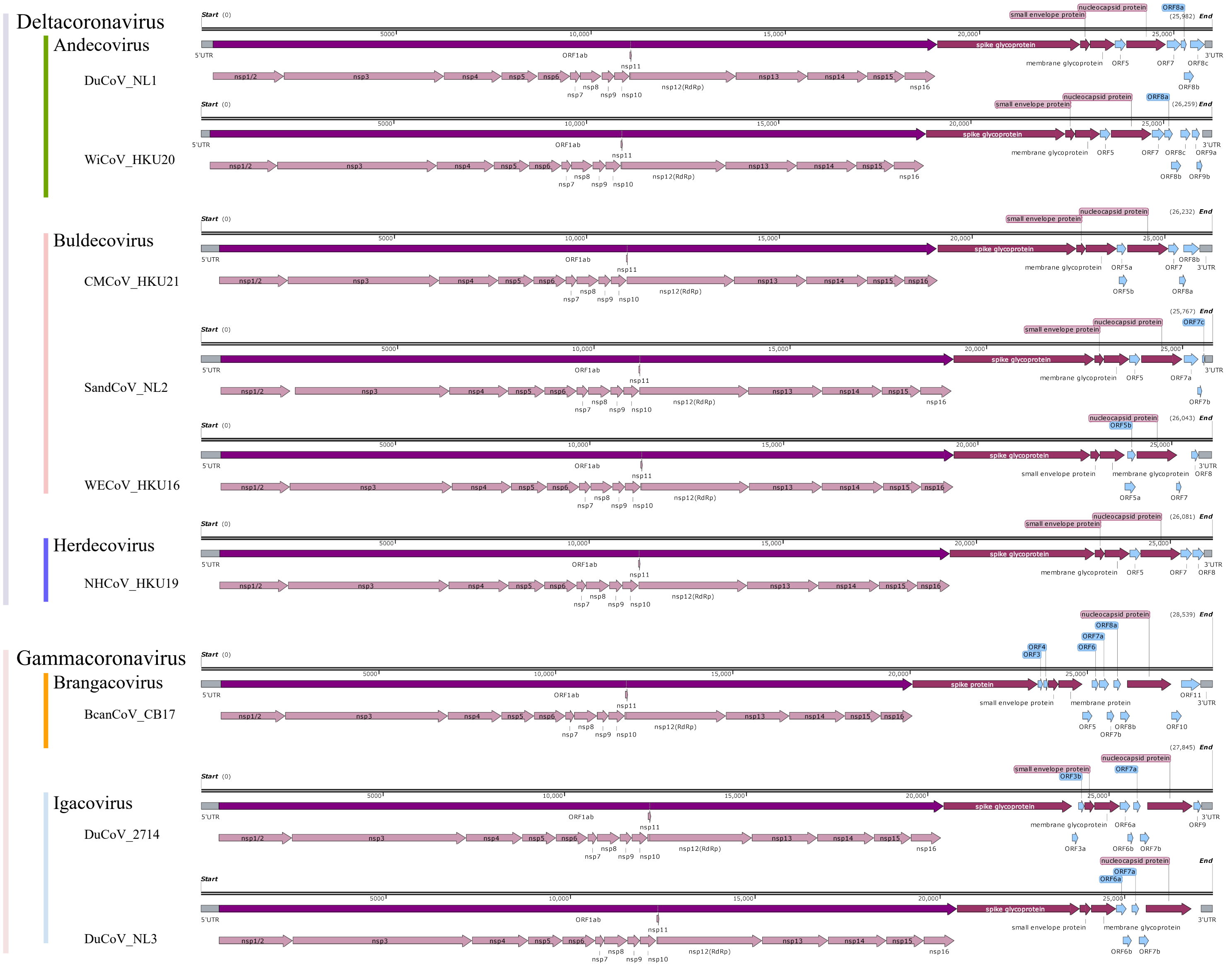

Supplementary Fig 9

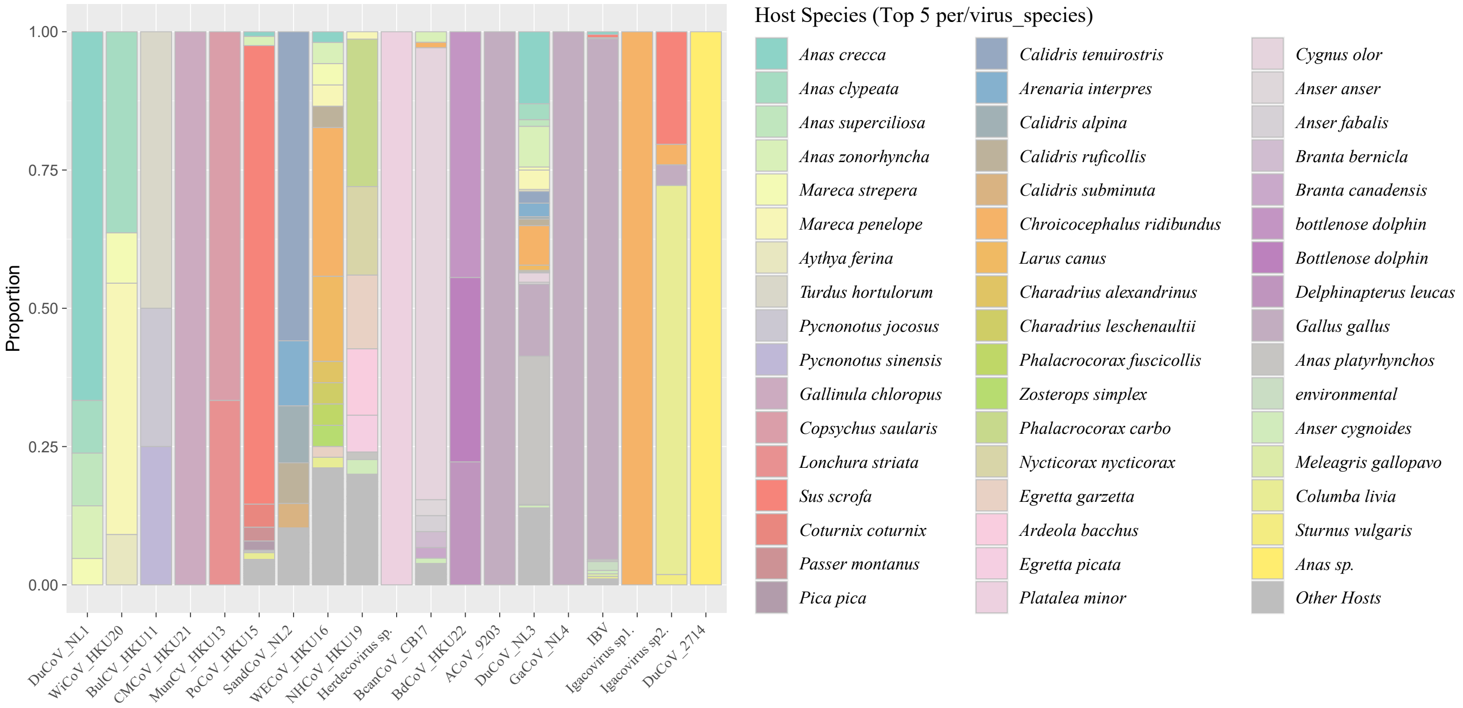

Supplementary Fig 10
